# Acute enhancement strategies for countermovement jump performance: a network meta-analysis of different resistance training protocols

**DOI:** 10.3389/fphys.2026.1729372

**Published:** 2026-01-30

**Authors:** Ying Zhou, Kaiming Wen, Yulong Sun

**Affiliations:** 1 College of Physical Education and Health, Guangxi Normal University, Guilin, Guangxi, China; 2 Hainan Medical University, Haikou, Hainan, China; 3 West England College of Health Sciences, Hainan Medical University, Haikou, Hainan, China; 4 Guangxi University School of Physical Education, Nanning, Guangxi Province, China

**Keywords:** deadlifts, flywheel training, leg presses, post-activation performance enhancement, post-activation potentiation, power, squats

## Abstract

**Background:**

Countermovement jump performance is the gold standard for assessing lower limb power, and even minor improvements can significantly enhance performance in sports such as basketball and volleyball. Post-activation potentiation (PAP) and post-activation performance enhancement (PAPE) are key mechanisms for acute performance enhancement, but the relative efficacy of different resistance training protocols (such as squats, deadlifts, flywheel training, and leg presses) remains unclear.

**Objective:**

To quantify and rank the acute potentiating effects of four resistance training protocols (conventional squats, flywheel squats, deadlifts, and leg presses) on countermovement jump performance through a network meta-analysis, and to explore the optimal load intensity and rest interval for the best resistance training modality.

**Methods:**

Six major databases were searched (up to May 2025), and 51 randomized controlled trials (involving 886 athletes) were included. A network meta-analysis within a frequentist framework was conducted, with standardized mean differences (SMD) and surface under the cumulative ranking curve (SUCRA) used to assess the efficacy ranking. Subgroup analyses were performed based on load intensity (≥85% 1RM for high intensity, <85% 1RM for moderate-low intensity) and rest interval (short: 0–4 min; medium: 5–7 min; long: ≥8 min).

**Results:**

A total of 51 studies were included. Flywheel training had the highest SUCRA value (95.8%), with a significant improvement in countermovement jump performance (SMD = 0.67, 95% CI: 0.22–1.12). This was followed by deadlifts (SUCRA = 62.4%, SMD = 0.28, 95% CI: 0.22%-0.78%) and back squats (SUCRA = 57.6%, SMD = 0.23, 95% CI: 0.03%-0.48%). Leg presses may have a negative impact on countermovement jump performance (SUCRA = 9.4%, SMD = −0.36, 95% CI: 1.18%-0.45%). For flywheel training, the best results were observed with moderate intensity (SMD = 0.92, 95% CI: 0.05%–1.80%) and medium rest intervals (SMD = 0.96, 95% CI: 0.04%–1.87%).

**Conclusion:**

Based on evidence of high quality level, Flywheel training is the best way to enhance acute countermovement jump performance. Regarding training parameters, while subgroup analyses point towards moderate intensity and 5–7 min of rest, these should be viewed as preliminary indicators due to wide confidence intervals and residual heterogeneity. While the conclusions for deadlifts and squats are based on less conclusive evidence, they are recommended as alternative options when a flywheel device is not available. If conditions do not permit, deadlifts can be considered as the next best option. However, the current evidence is insufficient to support the positive role of leg press in enhancing acute jumping ability.

## Graphical Abstract

**Figure:**
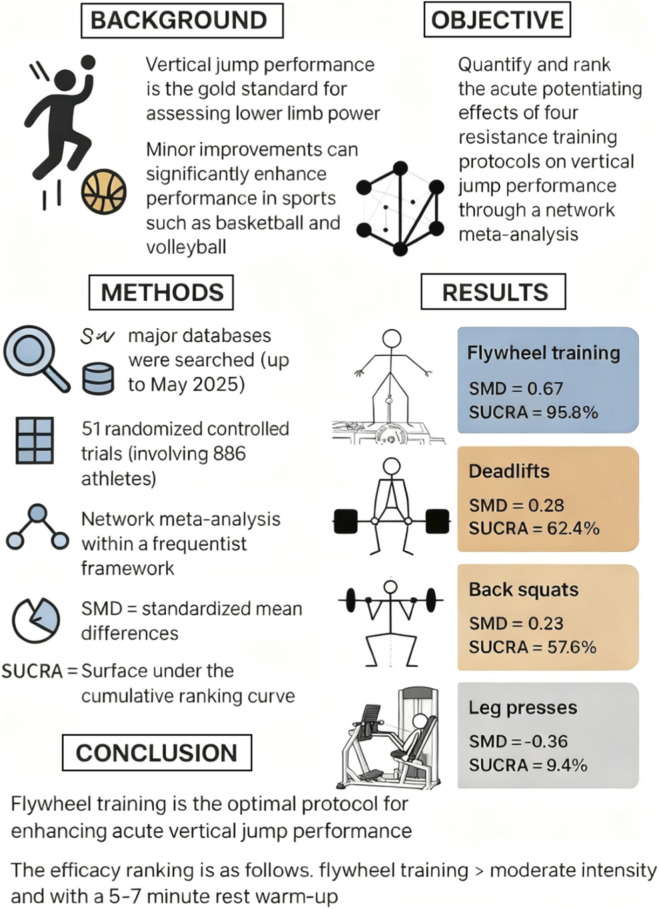
The graphical summary of this study.

## 1 Introduction

Explosive power is a key ability to enhance sports performance in most events (Boullosa et al., 2013; Carlock et al., 2004), and vertical jump performance is the gold standard for assessing lower limb vertical explosive power in sports populations, including Countermovement Jump (CMJ), Squat Jump (SJ), and Drop Jump (DJ) (Taylor et al., 2012), Minor improvements in vertical jump performance may translate into better results in jumping sports such as basketball and volleyball (Wilson et al., 2013).

In the 1970s and 1980s, Burke proposed the phenomenon of post-activation potentiation (PAP) (Burke et al., 1976), which specifically refers to the temporary increase in muscle contractile force after high-load (>85% 1RM) stimulation (Botelho and Cander, 1953; Ramsey and Street, 1941). In 2017, Cuenca-Fernandez et al. proposed a new concept—post-activation performance enhancement (PAPE), which refers to the brief improvement in subsequent sports performance caused by pre-load stimulation (Cuenca-Fernández et al., 2017; Boullosa et al., 2020). Although PAP and PAPE differ in their duration of action and physiological mechanisms, their biological goals are consistent—to optimize explosive power output through acute neuromuscular adaptation, thereby enhancing sports performance. Although PAP (primarily involving myosin light chain phosphorylation) and PAPE (associated with changes in muscle temperature, rheological properties, and neural drive) are mechanistically distinct, the new taxonomy proposed by Boullosa et al. (2013) suggests utilizing a broader term to encompass performance enhancements following a conditioning stimulus. In practical sports settings, where multiple mechanisms often co-occur and are difficult to fully isolate, adopting the term “Post-Activation Effect (PAE)” is of greater pragmatic significance. Accordingly, consistent with the perspective of Xu et al. (2025), the present study collectively refers to these two mechanisms as PAE to encompass their shared attributes in optimizing athletic performance.

Existing studies have shown that a variety of activation methods (such as electrical stimulation, resistance training, plyometric training, and sprint training) can induce PAE by enhancing neural impulses (Seitz and Haff, 2016; Creekmur et al., 2017; Arabatzi et al., 2014; Mccann and Flanagan, 2010; Till and Cooke, 2009). Tsoukos pointed out that the choice of exercise may affect the effectiveness of the conditioning stimulus, and future studies should directly compare various exercises, including deadlifts and squats, to determine their relative effects on the potentiation response (Arias et al., 2016). In previous reviews on PAE, the main activation method was resistance training, and most focused on a single training method (such as back squats) or pairwise comparisons, such as the differences in activation effects of different load intensities and rest intervals of barbell squats (Chen et al., 2023), whether the eccentric overload characteristics of flywheel training are superior to traditional concentric training (Xie et al., 2022), but they could not solve the core question of “which resistance training protocol is optimal for acute vertical jump enhancement?”

In view of this, this study for the first time integrates direct and indirect evidence to quantitatively compare the relative efficacy of four main resistance training protocols (conventional squats, flywheel squats, deadlifts, leg presses) and the control group, and to determine the ranking of different resistance training protocols in terms of acute potentiation effects on countermovement jump performance through network meta-analysis (NMA). Based on existing relevant literature, we defined the period within 20 min post-intervention as the acute enhancement phase for countermovement jump performance. In addition, based on load intensity, it is divided into high intensity (≥85% 1RM) and moderate-low intensity (<85% 1RM) (Beato et al., 2021; Wilson et al., 2013), and recovery time is divided into three time periods: short (0–4 min), medium (5–7 min), and long (≥8 min) (Xie et al., 2024; Seitz and Haff, 2016), Subgroup analyses are conducted for resistance training methods with significant improvement effects to further refine training elements and provide evidence-based basis for optimizing athletes' pre-competition warm-up design.

## 2 Methods

### 2.1 Protocol and registration

The protocol for this systematic review and network meta-analysis has been registered with PROSPERO (registration number: CRD420251173246). This study adheres to the PRISMA 2020 (Preferred Reporting Items for Systematic Reviews and Meta-Analyses) guidelines and the extension statement for network meta-analysis (PRISMA-NMA) (Page et al., 2021; Hutton et al., 2015).

### 2.2 Search strategy and study selection

Systematic searches were conducted in the PubMed, Web of Science, Cochrane (CENTRAL), Embase, Scopus, and EBSCOhost databases to identify randomized controlled trials published from the inception of the databases to May 29, 2025, that examined the effects of different resistance training protocols on athletic populations. Three reviewers independently searched and screened studies for eligibility, with disagreements resolved by consulting a fourth reviewer. Additionally, reference lists of included articles and relevant systematic reviews were hand-searched to identify potential eligible studies. The complete search strategy is detailed in the Supplementary Material.

### 2.3 Eligibility criteria

We assessed the eligibility of studies using the PICOS approach (Participants, Interventions, Comparators, Outcomes, and Study Design) (Liberati et al., 2009). Studies were included in the review if they met all the following criteria.

#### 2.3.1 Population

To maintain an adequate sample size while ensuring a baseline level of physical literacy, we specify that participants must have consistent resistance training experience (averaging at least two sessions per week), encompassing both recreational and professional athletes.

#### 2.3.2 Intervention

Based on the literature on potentiation effects, we categorized lower limb resistance exercises into four main types: conventional squats, flywheel squats, deadlifts, and leg presses, without distinguishing between static or dynamic slow movement patterns and movement speeds. Studies were included for review if they had any two or more of the above resistance training groups as experimental groups, or if they had only one of the above resistance training protocols as an experimental group but also included a control group. Furthermore, based on existing relevant literature, we defined the period within 20 min post-intervention as the acute enhancement phase for countermovement jump performance.

#### 2.3.3 Comparator

Control groups included no intervention (rest), low-intensity activity (e.g., slow walking, stretching), or routine warm-up training (the usual warm-up for specific training).

#### 2.3.4 Outcome

Height, peak power, and rate of force development of countermovement jumps (CMJ), squat jumps (SJ), and drop jumps (DJ) are commonly used as direct measures to determine whether performance has increased, decreased, or remained unchanged after training interventions (Suchomel et al., 2016). Therefore, studies included in this review reported at least one of the following indicators: height, peak power, flight time, take-off velocity, or other indicators reflecting vertical power during CMJ, DJ, or SJ.

#### 2.3.5 Study design

Randomized controlled trials.

### 2.4 Exclusion criteria

Confounding Interventions: Studies were excluded if the experimental protocol combined the specified resistance training with other physical or electrophysiological enhancement modalities, such as neuromuscular electrical stimulation (NMES), vibration training, pharmacological interventions, or specialized dietary controls.

Participants must not have performed high-intensity training or participated in official competitions within 24 h before or after the primary intervention to avoid the interference of cumulative fatigue on the PAPE (Post-Activation Performance Enhancement) response.

Inadequate Training Experience: Studies were excluded if the participants' resistance training experience did not meet the quantified criteria, specifically a training frequency of less than twice per week.

Non-acute Effect Studies: Research focusing on the chronic adaptation effects of long-term resistance training (>4 weeks) was excluded, as the primary focus is on the transient activation effects following a single stimulus.

Incomplete Data or Reporting: Studies were excluded if they failed to report key baseline jump data or lacked core statistics required for calculating Effect Sizes (e.g., Mean and Standard Deviation for SMD).

Non-randomized controlled trials (non-RCTs), conference abstracts, reviews, or non-peer-reviewed reports were also excluded.

The study did not report relevant indicators such as height, peak power, or rate of force development of countermovement jumps (CMJ), squat jumps (SJ), or drop jumps (DJ).

### 2.5 Data extraction

For each study that met the inclusion criteria, the research team independently collected the following key information using a pre-defined standardized form: basic study characteristics (e.g., first author’s name, year of publication, and country of the study), demographic characteristics of the participants (including age, sex, and sample size), detailed characteristics of the intervention (specific methods, training intensity, and number of training sets), and the primary outcome measures of the study. When a study included multiple experimental groups using the resistance training methods mentioned above, the research team selected the data from the group with the most significant test results for inclusion in the final analysis. Similarly, if a study had multiple time points of data recorded in the post-test phase, the research team selected the data with the most significant improvement for analysis. During the data extraction process, two independent researchers were responsible for the extraction, followed by a third researcher who verified and arbitrated the data. If the relevant data could not be found in the literature, the research team attempted to contact the corresponding author of the article three times within 3 weeks to obtain the required information.

### 2.6 Measures of treatment effect

In this meta-analysis, we assessed the treatment effect using the change in mean difference (Mean Difference, MD) and standard deviation (Standard Deviation, SD). If the original study did not directly provide the SD value, we estimated the SD based on the standard error (Standard Error), 95% confidence interval (Confidence Interval, CI), p-value, or t-statistic (Chandler et al., 2019). In this meta-analysis, we assessed the treatment effect using the change in mean difference (Mean Difference, MD) and standard deviation (Standard Deviation, SD). If the original study did not directly provide the SD value, we estimated the SD based on the standard error (Standard Error), 95% confidence interval (Confidence Interval, CI), p-value, or t-statistic (Chandler et al., 2019).

### 2.7 Quality assessment of evidence

We used the Cochrane Risk of Bias 2 tool for randomized trials (ROB 2 IRPG 2018) to conduct a comprehensive risk of bias assessment for the included trials, covering aspects such as random sequence generation, allocation concealment, blinding, missing outcome data, and selective outcome reporting (Sterne et al., 2019). For each study, if the risk of bias in all domains was rated as low, the overall risk of bias for that study was considered low (scored as 1); if at least one domain was rated as high risk, the overall risk of bias was considered high (scored as 3); in other cases, the risk of bias was considered to have some concerns (scored as 2). Two reviewers independently completed the risk of bias assessment, and any disagreements were resolved through discussion.

To detect small sample effects and publication bias, we constructed funnel plots for each direct comparison. Additionally, we used the CINeMA (Confidence in Network Meta-Analysis) framework to assess the certainty of the evidence across six key domains: within-study bias, reporting bias, indirectness, imprecision, heterogeneity, and inconsistency (Nikolakopoulou et al., 2020; Papakonstantinou et al., 2020). These domains evaluated the potential for systematic error within individual studies, the impact of selective reporting and publication bias, the relevance of the evidence to the research question, the uncertainty range of the effect estimates, the consistency of results across different studies, and the differences between direct and indirect evidence.

### 2.8 Statistical analysis

Notably, the current study collectively refers to PAP and PAPE as PAE, while functionally differentiating their respective contributions through a stratified analysis of rest intervals. Shorter intervals (0–4 min) primarily reflect the early interplay between PAP and fatigue, whereas moderate-to-long intervals (≥5 min) more accurately capture the delayed enhancement effects characteristic of PAPE. To systematically evaluate the geometric relationships of the acute potentiation effects of different resistance training interventions, we used the “networkplot” function in Stata software (version 15.0). A frequentist framework was adopted for the network meta-analysis, as it provides robust and computationally stable estimations for datasets of this scale. This approach also allows for direct evaluation of inconsistency through the node-splitting method. We used a random-effects model to fully account for heterogeneity between studies4. The heterogeneity parameter (tau^2) was estimated using the Restricted Maximum Likelihood (REML) method, which provides unbiased variance component estimates.

To ensure comparability of results, we used a consistent scoring standard or unit for each outcome measure in the analysis, selecting the standardized mean difference (SMD) as the primary effect size and calculating the corresponding 95% credible interval (CrI). Heterogeneity between studies was assessed according to previously published methods, measured by τ^2^ (Turner et al., 2012; Da Costa and Jüni, 2014) (low <0.04; low-moderate 0.04–0.16; moderate-high 0.16–0.36; high >0.36). To assess consistency between direct and indirect evidence within the network, we used the node-splitting method, detecting potential inconsistencies by comparing differences between direct and indirect effects. If the p-value was less than 0.05, it indicated significant inconsistency (Dias et al., 2013). We used the surface under the cumulative ranking curve (SUCRA) to rank the efficacy of the interventions. SUCRA quantifies the cumulative ranking probability of interventions across all comparisons, calculating the overall probability of an intervention being the best treatment option, thereby identifying the most effective exercise intervention. To further explore potential moderators that might affect treatment effects, we conducted meta-regression, analyzing potential moderators such as sex, load intensity, and rest intervals to explain the sources of heterogeneity (Mbuagbaw et al., 2017).

## 3 Results

### 3.1 Literature selection and study characteristics

**FIGURE 1:**
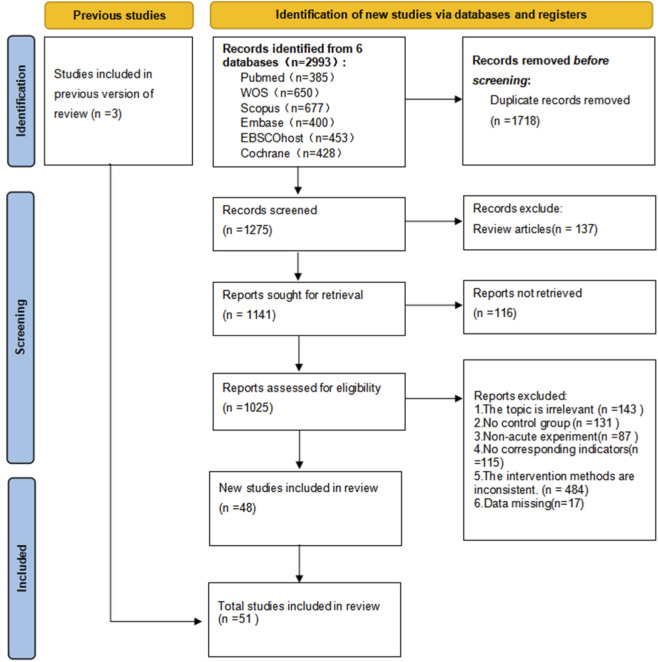
Flowchart of literature screening process.

A total of 2993 potentially relevant records were identified through the systematic search. After removing duplicates, 1275 articles remained for title and abstract screening. The authors reviewed the full text of 93 articles that met the criteria for full-text screening, and 48 articles were found to be eligible for inclusion. Ultimately, with the addition of three extra studies identified from other relevant reviews, a total of 51 studies were included in this review and meta-analysis, involving 886 participants. The complete process of screening and selection is shown in Figure 1, and the characteristics of the included studies are summarized in Table 1.

**TABLE 1:** Basic characteristics of the included studies.

Study	Author	Year	Sample size	Age	Intervention	Intensity	Intermission time	Gender	Outcome
1	Abade, E	2023	10	29.0 ± 6.4	DL	60%–85%1RM	15 min	Male	CMJ
2	Amiri-Khorasani, M	2024	32	24.5 ± 2.63	BQ	30%–50%1RM	2 min	Female	CMJ
3	Arias, J. C	2016	15	23.9 ± 4.2	DL	85%1RM	6 min	Male	CMJ
4	Baena-Raya, A	2023	16	23.5 ± 2	BQ	85%1RM	4 min	Male	CMJ
5	Bauer, P	2019	60	23.3 ± 3.3	BQ	60%1RM	3 min	Male	CMJ
6	Beato, M	2019	10	22 ± 2	FT, BQ	0.06 kg/m2	7 min	Male	CMJ
7	Villalon-Gasch, L	2020	11	22.6 ± 3.5	BQ	90%1RM	8 min	Female	CMJ
8	Chen, L. L	2024	18	23.6 ± 2.0	BQ, DL	3RM	4–8 min	Male	CMJ
9	Crum, A. J	2012	20	18–35	BQ	65%1RM	30 s	Male	Peak power
10	Cuevas-Aburto, J	2022	31	21.3 ± 2.3	BQ	10RM	10 min	Male	CMJ
11	do Carmo, E. C	2018	12	25.4 ± 3.6	BQ	5RM	4 min	Male	CMJ
12	Downey, R. J	2022	24	23.3 ± 4.4	BQ	70%1RM	3 min	Male\Female	CMJ
13	Faller, J. M	2023	14	Not reported	BQ	90%1RM	45 s	Male	CMJ
14	Fiorilli, G	2020	12	13.3 ± 0.7	FT	Unknow	5 min	Male	CMJ
15	Fletcher, I. M	2013	16	21.38 ± 0.5	BQ	90%1RM	4 min	Male	CMJ
16	Fontanetti, G	2025	10	20.6 ± 1.5	BQ	5RM	4 min	Male	CMJ
17	González-R, J. M	2009	24	21.6 ± 1.1	BQ	85%1RM	3 min	Male	CMJ
18	Hirayama, K	2014	14	19.9 ± 1.4	BQ	80%1RM	1 min	Male	CMJ
19	Jamaikon Carvalho	2020	15	24.9 ± 5.9	BQ	90%1RM	10 min	Male	CMJ
20	Jiang, X	2023	24	23.8 ± 0.7	BQ	80%1RM	8 min	Male	CMJ
21	Jones, P	2003	8	23.6 ± 3.4	BQ	85%1RM	13 min	Male	CMJ
22	Kannas, T. M	2024	20	21.2 ± 1.7	BQ	85%1RM	20 s	Male	CMJ
23	Masel, S	2024	15	22.9 ± 2.1	DL	80%1RM	90 s	Male	SJ
24	Krčmár, M	2015	11	22 ± 1.8	BQ	4RM	10 min	Male\Female	Power
25	Liu, Y	2024	21	Not reported	FT, BQ	80%1RM	6 min	Male	CMJ
26	Krzysztofik, M	2023	16	18–19	BQ	85%1RM	9 min	Male	CMJ
27	Tsoukos, A	2025	16	21.8 ± 1.2	FT, BQ	0.10 kg·m2	6 min	Male\Female	CMJ
28	Qi, H	2025	20	20.1 ± 2	FT	0.035 ± 0.01 kg·m2	8 min	Male	CMJ
29	Munger, C. N	2016	10	23.36 ± 3.8	DL	85%1RM	3 min	Male	Take-off speed
30	Piper, A. D	2020	13	20 ± 2	BQ	87%1RM	8 min	Male\Female	CMJ
31	Scott, D. J	2017	20	22.30 ± 2.9	DL, BQ	93%1RM	2 min	Not reported	CMJ
32	Spudic, D	2023	19	24.9 ± 2.6	FT, BQ	0.025–0.125 kg∙m2	1 min	Not reported	CMJ
33	Reardon, D	2014	11	25.2 ± 3.6	BQ	1RM	8 min	Male	CMJ
34	Scott, D. J	2018	20	22.4 ± 0.68	DL	70%1RM	30 s	Not reported	CMJ
35	Li, T	2024	8	23.3 ± 1.3	BQ	80%1RM	8 min	Female	CMJ
36	Masel, S	2022	12	23 ± 2	DL	80%1RM	90 s	Male	CMJ
37	Santos da Silva, V	2024	14	22.3 ± 4.0	BQ	90% 1RM	6 min	Female	CMJ
38	Krzysztofik, M	2021	16	24 ± 5	BQ	80% 1 RM	10 min	Female	CMJ
39	Shi, J	2024	13	Not reported	FT, BQ	0.1568 kg·m2	8 min	Male	CMJ
40	Zois, J	2015	10	23.3 ± 2.5	LP	5RM	15 min	Male	CMJ
41	Piqueras-S., F	2024	26	23.8 ± 4.4	BQ	75% 1RM	20 s	Male	CMJ
42	Sañudo, B	2020	28	23.5 ± 5.3	FT, BQ	90% 1RM	4–5 min	Male	CMJ
43	Tsolakis, C	2011	23	21.8 ± 3.7	LP	1RM	0 s	Male\Female	Peak power
44	Loturco, Irineu	2024	13	24.5 ± 4.7	FT	0.02, 0.008 kg·m2	5 min	Male	CMJ
45	Mola, J. N	2014	22	23 ± 4.5	BQ	75, 90, 100%3RM	16 min	Not reported	CMJ
46	Saez SV, E	2007	12	21–24	BQ	80%–85%1 RM	5 min	Male	CMJ
47	Villalon-Gasch, L	2022	11	22.6 ± 3.5	BQ	90% 1RM	8 min	Female	CMJ
48	Moreno-Pérez, V	2021	26	19.22 ± 4.2	LP	85%1RM	0 s	Male	CMJ
49	Sun, S	2024	16	22.3 ± 1.3	FT, BQ	80%1 RM	4 min	Male	CMJ
50	Timon, R	2019	16	21.8 ± 2.7	FT, BQ	924 g	8 min	Male\Female	SJ
51	Xie, H	2022	12	20.63 ± 1.3	FT, BQ	0.015–0.075 kg∙m2	12 min	Male	CMJ

BQ: back squat, FT: flywheel training, DL: deadlift, LP: leg press.

### 3.2 Risk of bias, certainty of evidence, and consistency

**FIGURE 2:**
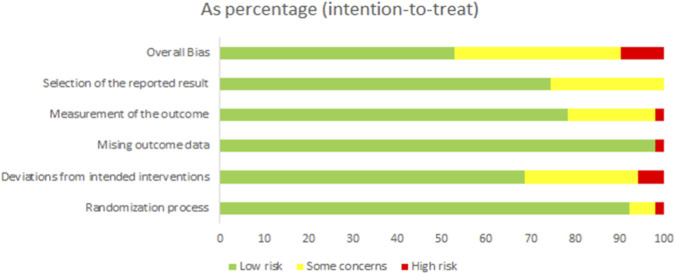
The risk of bias for each trial.

**FIGURE 3:**
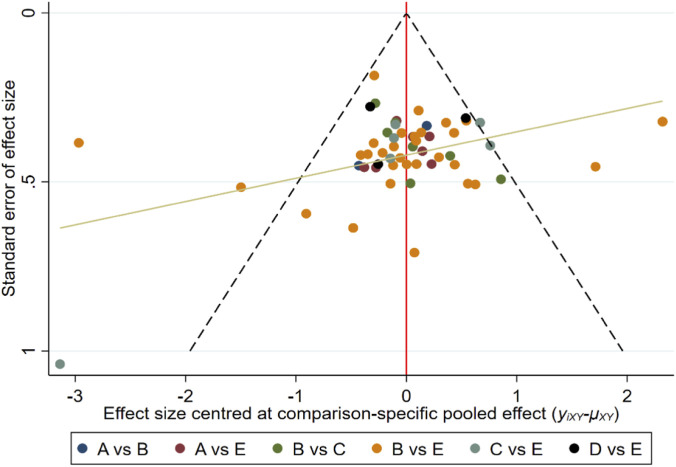
Funnel plot of vertical jump ability. Note: **(A)** deadlift, **(B)** BackSquat, **(C)** Flywheel training, **(D)** legpress, **(E)** Control group.

The risk of bias for each trial is shown in Figure 2. Overall, 27 studies (52.9%) were classified as low risk of bias, 19 studies (37.3%) as unclear risk of bias, and five studies (9.8%) as high risk of bias. The network model demonstrated excellent stability. In the consistency assessment (i.e., consistency between direct and indirect evidence), the node-splitting method revealed no local inconsistency (P > 0.05), and the global inconsistency test was also not significant (P = 0.98 > 0.05). The τ^2^ results indicated moderate-to-high heterogeneity within the network (τ^2^ = 0.36). After assessing the quality of evidence using the CINeMA framework, we found that, apart from the Control: Flywheel comparison having high-quality evidence, most pairwise comparisons had very low to moderate quality (Table 2). Additionally, no evidence of asymmetry was found in the funnel plot analysis, indicating no apparent publication bias (Figure 3).

**TABLE 2:** The results of the quality assessment of the included evidence in the CINeMA framework.

Comparison	Within-study bias	Reporting bias	Indirectness	Imprecision	Heterogeneity	Incoherence	Confidence rating
Back Squat: Control	No concerns	Low risk	No concerns	Major concerns	No concerns	No concerns	Low
Back Squat: Flywheel	Some concerns	Low risk	No concerns	No concerns	Major concerns	No concerns	Low
Back Squat: deadlift	No concerns	Low risk	No concerns	Major concerns	No concerns	No concerns	Low
Control: Flywheel	No concerns	Low risk	No concerns	No concerns	No concerns	No concerns	High
Control: deadlift	Some concerns	Low risk	No concerns	Major concerns	No concerns	No concerns	Low
Control group: leg press	Major concerns	Low risk	No concerns	Major concerns	No concerns	No concerns	Very low
Back Squat: leg press	Some concerns	Low risk	No concerns	No concerns	No concerns	No concerns	Moderate
deadlift: Flywheel	Some concerns	Low risk	No concerns	No concerns	No concerns	No concerns	Moderate
Flywheel: leg press	Some concerns	Low risk	No concerns	No concerns	No concerns	No concerns	Moderate
deadlift: leg press	Major concerns	Low risk	No concerns	Major concerns	No concerns	No concerns	Very low

### 3.3 Comparative analysis of different resistance training protocols on countermovement jump performance

**FIGURE 4:**
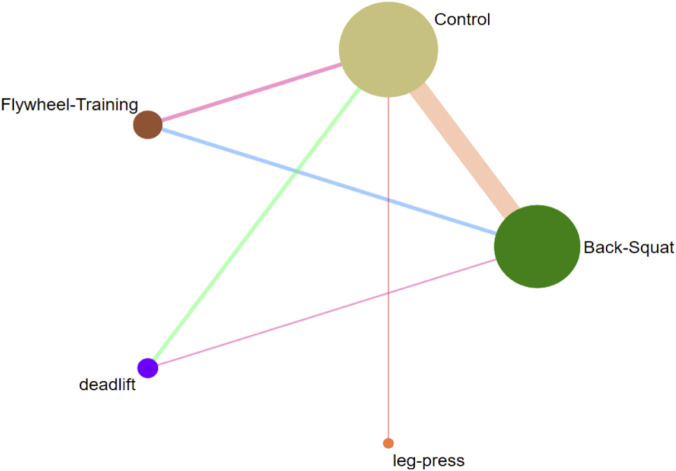
Network diagram of the effects of different resistance training on countermovement jump ability.

**FIGURE 5:**
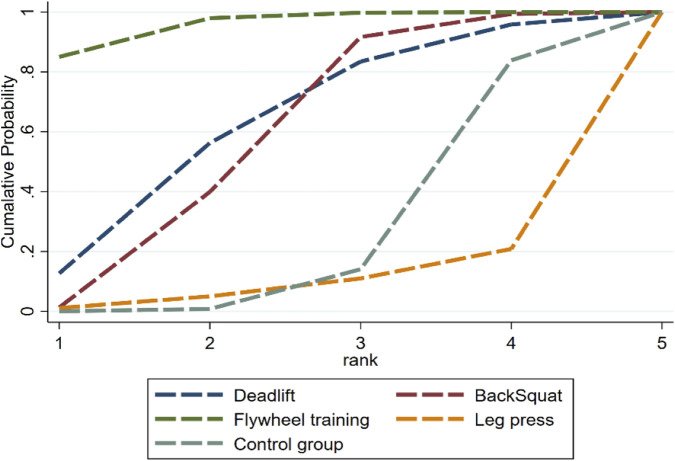
Cumulative probability graph of the effects of different resistance training on countermovement jump performance.

The network meta-analysis results (Figure 4; Table 3) showed that different resistance training protocols had varying effects on acute countermovement jump performance compared with the control group: Flywheel training had a significant positive effect (SMD = 0.67, 95% CI: 0.22–1.12; SUCRA 95.8%; high certainty of evidence). The effect of Back Squat was not significant (SMD = 0.23, 95% CI: 0.03 to 0.48; SUCRA 57.6%; low confidence of evidence). The effect of Deadlift was also not significant (SMD = 0.28, 95% CI: 0.22 to 0.78; SUCRA 62.4%; low confidence of evidence). However, the current evidence is insufficient to support the positive role of leg press in enhancing acute jumping ability (SMD = −0.36, 95% CI: 1.18 to 0.45; SUCRA 9.4%; low confidence of evidence). The ranking of the effects of different training protocols (Figure 5) was as follows: Flywheel training > Deadlift > Back Squat > Control group > Leg press. But these analyses revealed a wide confidence interval and residual heterogeneity. While the ranking suggests a hierarchy, it should be interpreted with caution as the comparisons between deadlifts, back squats, and leg presses are supported by low-to-very low certainty evidence. Therefore, the description of this analysis is merely exploratory.

**TABLE 3:** League table of network comparisons of the effects of different resistance training methods on countermovement jump performance.

Deadlift	−0.06 (−0.59,0.47)	0.39 (−0.27,1.04)	−0.64 (−1.60,0.31)	−0.28 (−0.78,0.22)
0.06 (−0.47,0.59) *	**Back squat**	**0.44 (0.00,0.88)**	−0.59 (−1.44,0.26)	−0.23 (−0.48,0.03)
−0.39 (−1.04,0.27) †	**−0.44 (-0.88,-0.00)** *	**Flywheel training**	**−1.03 (-1.96,-0.10)**	**−0.67 (-1.12,-0.22)**
0.64 (−0.31,1.60) *	0.59 (−0.26,1.44) †	**1.03 (0.10,1.96)** †	**Leg press**	0.36 (−0.45,1.18)
0.28 (−0.22,0.78) *	0.23 (−0.03,0.48) *	**0.67 (0.22,1.12)**‡	−0.36 (−1.18,0.45) *	**Control group**

The table presents the network meta-analysis of the effects of different resistance training protocols on countermovement jump performance, with all effect sizes expressed as standardized mean differences (SMD) and 95% credible intervals (CrI). Cells in bold indicate significant results. According to the CINeMA (Confidence in Network Meta-Analysis) framework, the credibility of the evidence for each comparison is included in the league table, with * indicating low confidence, † indicating moderate confidence, and ‡ indicating high confidence. Bold numbers indicate statistically significant differences. Bold text denotes the intervention.

### 3.4 Subgroup analysis: flywheel training

For flywheel training, which had a significant positive effect, we conducted further subgroup analyses on load intensity (Table 4) and rest interval (Table 5) to explore the optimal intensity and rest interval. The results showed that flywheel training at moderate intensity achieved a significant effect (SMD = 0.92, 95% CI: 0.05–1.80), while no significant differences were found for low and high intensities. Similarly, flywheel training after a medium rest interval achieved a significant effect (SMD = 0.96, 95% CI: 0.04–1.87), while no significant differences were found for long and short rest intervals. Preliminary evidence suggests that a training protocol of moderate intensity combined with 5–7 min of rest may provide beneficial potentiating effects. However, these analyses revealed a wide confidence interval and residual heterogeneity. Therefore, the description of this analysis is merely exploratory.

**TABLE 4:** League table of flywheel training divided into subgroups by intensity.

Flywheel (Moderate to low intensity)	−0.44 (−2.13,1.24)	−0.92 (−1.80,-0.05)
0.44 (−1.24,2.13)	**Flywheel** **(High intensity)**	−0.48 (−1.92,0.96)
**0.92 (0.05,1.80)**	0.48 (−0.96,1.92)	**Control**

High-intensity refers to ≥85% 1RM, while moderate-low intensity refers to <85% 1RM. Bold numbers indicate statistically significant differences. Bold text denotes the intervention.

**TABLE 5:** League table of flywheel training divided into subgroups by Intermission time.

Flywheel (Long interval)	0.44 (−1.00,1.87)	−0.25 (−2.58,2.08)	−0.52 (−1.63,0.59)
−0.44 (−1.87,1.00)	**Flywheel(Medium interval)**	−0.69 (−2.93,1.56)	−0.96 (−1.87,-0.04)
0.25 (−2.08,2.58)	0.69 (−1.56,2.93)	**Flywheel** **(Short interval)**	−0.27 (−2.32,1.78)
0.52 (−0.59,1.63)	**0.96 (0.04,1.87)**	0.27 (−1.78,2.32)	**Contrl**

Short interval refer to 0–4 min, medium interval refer to 5–7 min, and long interval refer to 8 min or more. Bold numbers indicate statistically significant differences. Bold text denotes the intervention.

### 3.5 Sensitivity analyses and meta-regressions

We conducted subgroup analyses to explore potential sources of heterogeneity in the outcome measures. After independent double-checking of data extraction, no errors were found. Subgroup analyses by training intensity, rest interval, and sex did not reveal a significant reduction in heterogeneity, except for the high-intensity group, where heterogeneity was significantly reduced (I^2^ decreased from 78% to 23.3%), suggesting that load intensity might be a potential source of heterogeneity. While these parameters provide a practical framework, the remaining heterogeneity suggests that individual responses to flywheel PAPE may still be influenced by unmeasured factors. However, meta-regression did not find a significant association between training intensity and effect size (P = 0.16). This inconsistency may be related to other unmeasured homogeneous factors within the high-intensity subgroup (such as age distribution), which requires further research for validation.

We performed additional subgroup analyses and meta-regressions based on three key dimensions: professional status, baseline strength level, and training experience. Regarding professional status, although subgroup stratification did not lead to a significantattenuation of within-group heterogeneity (Professional: I^2^ = 80.1%; Recreational: I^2^ = 76.2%), meta-regression identified it as a significant moderator (p < 0.05) that accounted for 8.29% of thebetween-study variance (*R* ^2^ = 8.29%).

When stratifying by baseline strength level, the heterogeneity in the “high-strength” subgroup wasmarkedly reduced to 27.8% (compared to the overall 83%), whereas the “low-strength” groupremained highly heterogeneous (I^2^ = 77.1%). Despite this within-group reduction, meta-regressionfailed to confirm strength level as a significant global moderator (p > 0.05, *R* ^2^ = −2.8%).

Finally, while training years did not show a clear trend in reduction across subgroups (63% and 78.6% for <3 and >3 years, respectively), meta-regression revealed that training experience was apotent source of heterogeneity (p < 0.05), explaining 21.2% of the total variance. These findings suggest that while professional status and training experience significantly moderatethe PAE response, substantial residual heterogeneity persists, likely reflecting the complexmultifactorial nature of post-activation performance enhancement in athletic populations.

**FIGURE 6:**
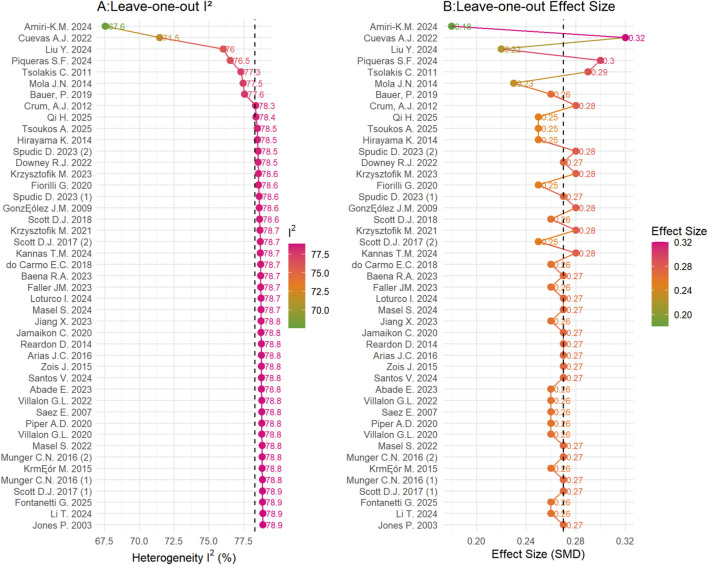
Heterogeneity and pooled effect size after the exclusion of each individual study. **(A)** is the leave-one-out analysis of I^2^, and **(B)** is the leave-one-out analysis of effect size, with green indicating low values and red indicating high values.

To verify the robustness of the results and address potential selection bias, we conducted a sensitivity analysis. To ensure the robustness of our conclusions without diluting the specific temporal characteristics of the potentiating effect, we performed this analysis using the last post-intervention time point reported in each study (rather than the peak significant value). The rationale for selecting the last time point is based on the physiological distinction between post-activation potentiation (PAP) and post-activation performance enhancement (PAPE). While immediate effects (0–3 min) are often influenced by acute fatigue, later time points (typically >6 min) better reflect a more stable state of post-activation performance enhancement (PAPE). The results indicate (Table 6) that even when using the most conservative “last time point” data, the efficacy ranking remained consistent (flywheel > deadlift > back squat) and the effect sizes and their confidence intervals showed only minimal variation., supporting the robustness of our main conclusions. Additionally, a sensitivity analysis was performed using the leave-one-out method, where each study was systematically excluded to observe the resulting changes in I^2^ (Figure 6A) and effect size (Figure 6B). The results demonstrated that the pooled effect size remained stable at approximately 0.27, and I^2^ hovered around 78.3%, further confirming the robustness of our findings.

**TABLE 6:** League table presenting the results of the sensitivity analysis.

leg press	−0.54 (−1.40; 0.31)	−0.66 (−1.62; 0.31)	−1.05 (−1.98; −0.11)	−0.37 (−1.19; 0.45)
−0.54 (−1.40; 0.31)	**Back squat**	−0.11 (−0.65; 0.42)	−0.50 (−0.94; −0.06)	0.17 (−0.08; 0.43)
−0.66 (−1.62; 0.31)	−0.11 (−0.65; 0.42)	**deadlift**	−1.05 (−1.98; −0.11)	0.29 (−0.22; 0.79)
−1.05 (−1.98; −0.11)	−0.50 (−0.94; −0.06)	−0.39 (−1.05; 0.27)	**Flywheel training**	0.68 (0.23; 1.12)
−0.37 (−1.19; 0.45)	0.17 (−0.08; 0.43)	0.29 (−0.22; 0.79)	0.68 (0.23; 1.12)	**Control group**

Bold numbers indicate statistically significant differences. Bold text denotes the intervention.

To address the potential interference of different jump types on the efficacy ranking, we conducted a specific sensitivity analysis by excluding the two studies that utilized Squat Jump (SJ) as the outcome measure (Please refer to Supplementary Appendix Figures 7–12 in Appendix 7 of the Supplementary Material). The results showed that the efficacy ranking (Flywheel training > Deadlifts > Back squats > Control group > Leg presses) and the effect sizes remained consistent with the primary analysis, confirming the robustness of the findings for Countermovement Jump (CMJ) performance.

## 4 Discussion

### 4.1 Comparative analysis of the acute effects of different resistance training protocols on vertical jump performance in athletes

This study, for the first time, quantitatively compared the acute potentiating effects of four resistance training protocols on countermovement jump performance through a network meta-analysis.

The core findings are summarized as follows:

The superiority of Flywheel training is particularly evident in CMJ performance (SMD = 0.67, 95% CI: 0.22–1.12), with a SUCRA value of 95.8% and high-quality evidence (CINeMA rating: High). This finding indicates the unique advantage of eccentric overload in enhancing acute countermovement jump performance—flywheel resistance training is indeed quite different from muscle action patterns in elastic training modes, as it does not rely on the same mechanics (e.g., parallel or serial elastic components) and neural factors (e.g., stretch reflexes) (Xie et al., 2022), Instead, it depends on the rotational inertia generated by the flywheel, which results in a greater eccentric load than that produced by traditional resistance training, This can be attributed to the eccentric overload mechanism of flywheel devices, which enhances the stretch-shortening cycle (SSC) by increasing neuromuscular activation during the eccentric phase (Maroto-Izquierdo et al., 2017). Since CMJ is an SSC-dominant movement, the physiological adaptations induced by flywheel training are highly specific to this jump type. Studies have pointed out that prolonging the duration of muscle eccentric contraction can significantly improve strength performance (Martinez-Aranda and Fernandez-Gonzalo, 2017), Traditional barbell training exhibits a constant resistance pattern at different intensities. In contrast, flywheel devices utilize the principle of flywheel kinetic energy accumulation (Nuñez et al., 2019), When inertia increases, peak concentric velocity, peak eccentric velocity, mean concentric velocity, and mean eccentric velocity all tend to decrease, while the ratio of peak eccentric power to peak concentric power correspondingly increases (Mcerlain-Naylor and Beato, 2021; Gonzalo-Skok et al., 2017). The physiological benefits achieved by enhancing the eccentric phase include increased neuromuscular activation levels, optimized postural control capabilities, improved muscle coordination, and ultimately enhanced explosive power performance and reduced sprint times (Norrbrand et al., 2010). Tous-Fajardo et al. monitored muscle activity in football players using flywheel devices through surface electromyography, and their results showed that a single session of flywheel eccentric training elicited higher overall electromyographic activity levels than other strength training methods, indicating that flywheel training can promote more significant neuromuscular adaptive adjustments (Tous-Fajardo et al., 2006). Therefore, flywheel training optimizes neuromuscular adaptation through its unique eccentric overload mechanism and has become the most effective protocol for enhancing acute countermovement jump performance, providing athletes with a scientifically sound choice for pre-competition warm-ups based on biomechanical evidence.

Deadlift and back squat, although not statistically significant (SMD = 0.28 and 0.23, respectively), ranked second (62.4%) and third (57.6%) in the SUCRA ranking, suggesting that they still have practical value. Experiments by Arias et al. (2016), Till and Cooke (2009) and Masel and Maciejczyk (2024) have shown that deadlifts produce better performance enhancement effects than traditional squats, but neither enhancement effect was significant, which is consistent with the results of this study. The reason may be that in Arias’ study, there was only a 2-min interval between the intervention and the jump test, which may not have been sufficient for the body to recover and eliminate fatigue. In Till’s experiment, the countermovement jump test was conducted after a 20-m sprint, which may have further exacerbated fatigue and thus weakened the potentiation effect.

Based on the conclusions of this study, leg press may have a negative impact on countermovement jump performance. However, the confidence interval includes zero and the certainty of the evidence is low. Leg press may have a negative impact on countermovement jump performance (SMD = −0.36, SUCRA 9.4%). Tsolakis (Tsolakis et al., 2011) found in his study that in male fencers, peak leg strength output decreased after leg press training, while there was no change in the performance of female fencers. Therefore, he speculated that stronger subjects may experience a short-term decline in leg strength after leg press training, and his study suggests that strength level may be a factor affecting PAE. Although studies have shown that individuals with higher levels of performance may have better muscle activation rates, these individuals may also produce greater and more persistent fatigue. As the primary studies failed to stratify participants based on their strength levels, it remains uncertain whether the leg press exclusively exerts fatigue-suppressing effects in high-level athletes. Consequently, there is insufficient evidence to support specific recommendations for leg press training. Fatigue takes the lead, resulting in performance decline rather than improvement (Hamada et al., 2003). Furthermore, the leg press protocols employed in this study incorporated either very brief or no rest intervals. This may have counteracted both fatigue and the potentiating effect, ultimately leading to diminished countermovement jump performance. Following short-term high-intensity exercise priming, muscles enter a state characterized by the coexistence of fatigue and potentiation. Consequently, subsequent countermovement jump performance is dependent on the interaction between these two opposing factors and the rate of recovery following the conditioning activity (Anthony and Bishop, 2009). It should be noted that squats can effectively activate the core muscle groups involved in the countermovement jump movement, while leg press movements have lower activation rates of core muscles (Prieske et al., 2015), which may also be one of the reasons for the decline in countermovement jump performance after leg press training. In addition, performing countermovement jump movements requires the coordinated effort of knee, hip, ankle, and trunk muscle strength. The target muscle group of leg press is leg muscles (Wirth et al., 2016), Compared with flywheel, squat, and deadlift movements, leg press lacks stimulation of the gluteal muscles, resulting in poorer activation effects.

Although flywheel training is effective, its equipment is heavy and inconvenient to transport, expensive, and requires athletes to possess advanced operational skills (eccentric control), which explains why the squat remains the mainstream.

### 4.2 Subgroup exploration of the effectiveness of flywheel training

In this study, subgroup analyses were conducted on the training intensity and rest intervals of Flywheel Training to further explore its practical value. However, these analyses exhibited wide confidence intervals and residual heterogeneity. Therefore, the following descriptions of this analysis are presented as exploratory in nature. Load intensity. Although PAE is generally believed to be caused by high-intensity loads, there is evidence that it can also be induced by more moderate loads of 60%–85% 1RM (Wilson et al., 2013; Baker and Newton, 2005; Smilios et al., 2005). Scott believes that moderate-intensity loads combined with shorter rest intervals, equivalent to heavy resistance load stimulation, may be a more practical activation strategy for inducing PAE (Scott et al., 2018). Tesch also emphasized that under the same load, the muscle stimulation during flywheel training is greater than that of other resistance training, so moderate-intensity flywheel training can achieve the high-intensity level of traditional resistance training (Tesch et al., 2017).

Rest interval. Previous studies have shown that the rest interval between conditioning activation and testing is the most important factor in inducing explosive countermovement jump enhancement (Dobbs et al., 2019). Preliminary evidence suggests that a training protocol of moderate intensity combined with 5–7 min of rest may provide beneficial potentiating effects. Flywheel training yielded significant effects at rest intervals of 5–7 min, which aligns with the characteristic time window for PAPE. This suggests that the protocol enhances jump performance primarily through PAPE mechanisms—such as increased neural recruitment or elevated muscle temperature—rather than solely through transient PAP. Longer recovery times (4–8 min) produce better PAE effects than shorter recovery times (2–3 min), although there are individual differences (Xie et al., 2022; Seitz et al., 2014). Kannas' experiment showed that a single session of eccentric squat jumps with a short rest interval may not effectively enhance jumping ability. He speculated that a short rest interval seems insufficient to produce a potentiation effect because fatigue may dominate during this period, suppressing the emergence of the potentiation effect (Kannas et al., 2024). An appropriate inter-set recovery time can promptly eliminate the fatigue generated by flywheel training. When the working muscles have partially recovered but still have a potentiation effect, there is an opportunity to improve performance and thereby produce a PAE effect (Docherty et al., 2004; Tillin and Bishop, 2009), Tsoukos proposed that total force impulse, rather than inertia itself, is an important variable affecting PAE, highlighting the importance of considering total workload and rest intervals when designing flywheel training programs (Tsoukos et al., 2025). Therefore, regardless of the inertial load, coaches should focus on matching force impulses to optimize PAE, and controlling rest intervals is particularly important.

## 5 Conclusion

Based on evidence of high quality level, Flywheel training is the best way to enhance acute countermovement jump performance. Regarding training parameters, while subgroup analyses point towards moderate intensity and 5–7 min of rest, these should be viewed as preliminary indicators due to wide confidence intervals and residual heterogeneity. While the conclusions for deadlifts and squats are based on less conclusive evidence, they are recommended as alternative options when a flywheel device is not available. Therefore, in athlete training or pre-competition warm-ups, flywheel training is the first choice. If conditions do not permit, deadlifts can be considered as the next best option. However, the current evidence is insufficient to support the positive role of leg press in enhancing acute jumping ability.

## 6 Limitations, interpretation of findings, and future directions

While this network meta-analysis provides the first quantitative comparison of four resistance training protocols for acutely enhancing countermovement jump performance, several limitations should be considered when interpreting the results. More importantly, these limitations illuminate promising pathways for future research.

### 6.1 Specificity of jumping assessment

The primary outcome measure in over 90% of the included studies was the countermovement jump (CMJ). While the CMJ is a widely accepted and ecologically valid test for lower limb power, the lack of independent analysis of other jump types, such as the squat jump (SJ) and drop jump (DJ), may limit the generalizability of our conclusions. The SJ, which eliminates the stretch-shortening cycle, and the DJ, which emphasizes reactive strength, may respond differently to various post-activation enhancement protocols. Therefore, the current findings are most directly applicable to athletic contexts where CMJ performance is paramount. Future studies should directly compare the acute effects of these resistance training modalities on a battery of jump tests to determine if the efficacy ranking (Flywheel > Deadlift > Back Squat) holds across different neuromuscular performance metrics.

### 6.2 Heterogeneity and unexplored moderators

The significant population heterogeneity across the 51 included studies represents a major limitation of this network meta-analysis. We observed a moderate-to-high degree of heterogeneity (τ^2^ = 0.36) across the included studies. Although our subgroup analysis suggested that load intensity might be a potential source, this was not confirmed by meta-regression (P = 0.16). This indicates the presence of other, unmeasured confounding factors that influence the PAE response. There was a broad variance in participant demographics, including age (ranging from adolescents to adults over 30), sex distribution, and specific sports disciplines. Furthermore, while all participants were described as having athletic or resistance training experience, their precise training status—such as years of specialized experience, weekly training frequency, and baseline strength levels—was not uniformly quantified across all trials. These factors are known to critically modulate the PAE response, as stronger or more experienced athletes may exhibit different potentiation-fatigue profiles compared to less trained individuals. The lack of individual participant data precludes a more granular analysis of how these biological and professional variables interact with different training protocols, necessitating caution when generalizing the current hierarchy of interventions to specific athletic populations. The ranking of the four activation measures obtained in this study is based solely on the probability ranking of the existing evidence, and is not an absolute conclusion. To address this, we strongly recommend that future research on PAE adopts standardized reporting guidelines that include detailed individual characteristics. Furthermore, the application of Individual Participant Data (IPD) network meta-analysis would be a powerful next step, allowing for a more precise exploration of these individual-level moderators and a reduction in heterogeneity.

A significant limitation of this study lies in the absence of key baseline information across some of the included studies, which directly impacts the precision of our heterogeneity assessment. Despite conducting an exhaustive data retrieval process and attempting to contact the original authors, several studies failed to report critical participant characteristics such as precise age, sex distribution, or specific sports background (e.g., basketball, volleyball, or weightlifting). Within the research field of post-activation effects (PAE), these factors are considered pivotal confounding variables. Furthermore, the lack of baseline data restricted our ability to perform more in-depth subgroup analyses or meta-regressions, potentially masking the true sources of the moderate-to-high heterogeneity (τ^2^ = 0.36) observed within the network. Due to the inability to quantify participants' ‘training status'—such as maximal strength levels or years of specialized training—it remains difficult to determine the robustness of the current efficacy ranking across athletes of varying levels.

### 6.3 Interpretation of the quality of evidence levels

Flywheel training is the most reliable recommendation for acute CMJ enhancement, supported by high-quality evidence. However,the current evidence is insufficient to support the positive role of leg press in enhancing acute jumping ability. But the wide confidence interval (95% CI: 1.18 to 0.45) and low certainty of evidence suggest that the impact of leg press remains uncertain. This ambiguity highlights a critical gap in the literature. We call for targeted, high-quality RCTs with adequate sample sizes to conclusively determine the effect of leg press on acute power output. The ‘Low' confidence rating for the deadlift vs. back squat comparison stems from imprecision and within-study bias. Consequently, while deadlifts currently show a higher SUCRA value, the statistical gap is narrow and lacks high-quality support, meaning their relative positions in the efficacy hierarchy could be interchanged as more high-quality randomized controlled trials emerge.

### 6.4 Future research directions

Due to inconsistent reporting in the primary literature (e.g., missing data regarding sex, 1RM, and sport-specific distribution), future PAE studies should strictly adhere to the CONSORT statement or domain-specific reporting guidelines in sports science. Such adherence is essential to enhance the precision and granularity of future meta-analytic evidence.
