# Supplementary material for: Acute enhancement strategies for countermovement jump performance: a network meta-analysis of different resistance training protocols
Source: Front Physiol. 2026 Jan 30;17:1729372. doi: 10.3389/fphys.2026.1729372 (PMC12902682; doi:10.3389/fphys.2026.1729372)
Supplement: Supplementary file 2 [file Supplementaryfile1.docx]

| Database | Web of Science (all database) | |
| --- | --- | --- |
| Search Date | May 29th, 2025 | |
| Search Period | From the inception of database to May 29th, 2025 | |
| No. | Search strategy | Literatures retrieved |
| #1 | TS=("Squat" or "Barbell Squat" or "Back Squat" or "Low Bar Squat" or "High Bar Squat" or "Complex training" or "Contrast training" or " Hybrid Power Training" or "Flywheel eccentric training" or "Flywheel inertial training" or "Flywheel training" or "Flywheel resistance training" or "Inertia Flywheel" or "Eccentric overload" or "Isoinertial" or "deadlift" or "dead-lift" or "Conventional deadlift" or "Sumo deadlift" or "Romanian deadlift" or "Hex bar deadlift" or "Trap bar deadlift" or "Barbell deadlift" or "Hip hinge exercise" or "Weightlifting deadlift" or "leg press" or "LP" or "Leg-press" or "Seated leg press" or "45-degree leg press" or "Horizontal leg press" or "Vertical leg press" or "Pneumatic leg press" or "Machine leg press") | **84038** |
| #2 | TS=("Acute responses" or "Acute Effects" or "post-activation potentiation" or "post-activation performance enhancement" or "Postactivation Potentiation" or "postactivation performance enhancement" or "PAPE" or "PAP" or "Warm-up") | **53867** |
| #3 | AB=(“equilibrium” or “balance” or “functional reach” or “postur” or “dynamic postural control” or “gait” or “locomotion” or “walking” or “mobility” or “treadmill gait” or “ambulation” or “Stride Length” or “Stride Cadence” or “Gait Speed” or “Gait velocity” or “walking speed” or "activities-specific balancing confidence scale" OR "Unified Parkinson's Disease Rating Scale part III" OR "ABC" OR "UPDRS3-III") | **3026329** |
| #4 | #3 AND #2 AND #1 | **650** |

| Database | PubMed | | | |
| --- | --- | --- | --- | --- |
| Search Date | May 29th, 2025 | | | |
| Search Period | From the inception of database to May 29th, 2025 | | | |
| No. | Search strategy | | | Literatures retrieved |
| #1 | "Squat"[Title/Abstract] OR "Barbell Squat"[Title/Abstract] OR "Back Squat"[Title/Abstract] OR "Low Bar Squat"[Title/Abstract] OR "Complex training"[Title/Abstract] OR "Contrast training"[Title/Abstract] OR "Flywheel inertial training"[Title/Abstract] OR "Flywheel training"[Title/Abstract] OR "Flywheel resistance training"[Title/Abstract] OR "Eccentric overload"[Title/Abstract] OR "Isoinertial"[Title/Abstract] OR "deadlift"[Title/Abstract] OR "dead-lift"[Title/Abstract] OR "Conventional deadlift"[Title/Abstract] OR "Sumo deadlift"[Title/Abstract] OR "Romanian deadlift"[Title/Abstract] OR "Hex bar deadlift"[Title/Abstract] OR "Trap bar deadlift"[Title/Abstract] OR "Barbell deadlift"[Title/Abstract] OR "Leg-press"[Title/Abstract] OR "LP"[Title/Abstract] OR "Leg-press"[Title/Abstract] OR "Seated leg press"[Title/Abstract] OR "45-degree leg press"[Title/Abstract] OR "Horizontal leg press"[Title/Abstract] OR "Pneumatic leg press"[Title/Abstract] OR "Machine leg press"[Title/Abstract] | | | **42118** |
| #2 | "Acute responses"[Title/Abstract] OR "Acute Effects"[Title/Abstract] OR "post-activation potentiation"[Title/Abstract] OR "post-activation performance enhancement"[Title/Abstract] OR "Postactivation Potentiation"[Title/Abstract] OR "postactivation performance enhancement"[Title/Abstract] OR "PAPE"[Title/Abstract] OR "PAP"[Title/Abstract] OR "Warm-up"[Title/Abstract] | | | **44779** |
| #3 | "Jump"[Title/Abstract] OR "Counter Movement Jump"[Title/Abstract] OR "CMJ"[Title/Abstract] OR "Vertical Jump"[Title/Abstract] OR "Squat Jump"[Title/Abstract] OR "Drop Jump"[Title/Abstract] OR "Abalakov Jump"[Title/Abstract] OR "Standing long jump"[Title/Abstract] OR "Standing broad jump"[Title/Abstract] OR "Power"[Title/Abstract] OR "Maximum Output"[Title/Abstract] OR "Maximum Output"[Title/Abstract] OR "Peak Strength"[Title/Abstract] OR "Peak Output"[Title/Abstract] OR "Muscle power"[Title/Abstract] | | | **485,181** |
| #4 | #3 AND #2 AND #1 | | | **385** |
| Database | | EBSCOhost | | |
| Search Date | | May 29th, 2025 | | |
| Search Period | | From the inception of database to May 29th, 2025 | | |
| No. | | Search strategy | Literatures retrieved | |
| #1 | | AB ("Squat" or "Barbell Squat" or "Back Squat" or "Low Bar Squat" or "High Bar Squat" or "Complex training" or "Contrast training" or " Hybrid Power Training" or "Flywheel eccentric training" or "Flywheel inertial training" or "Flywheel training" or "Flywheel resistance training" or "Inertia Flywheel" or "Eccentric overload" or "Isoinertial" or "deadlift" or "dead-lift" or "Conventional deadlift" or "Sumo deadlift" or "Romanian deadlift" or "Hex bar deadlift" or "Trap bar deadlift" or "Barbell deadlift" or "Hip hinge exercise" or "Weightlifting deadlift" or "leg press" or "LP" or "Leg-press" or "Seated leg press" or "45-degree leg press" or "Horizontal leg press" or "Vertical leg press" or "Pneumatic leg press" or "Machine leg press") | **204,643** | |
| #2 | | AB ("Acute responses" or "Acute Effects" or "post-activation potentiation" or "post-activation performance enhancement" or "Postactivation Potentiation" or "postactivation performance enhancement" or "PAPE" or "PAP" or "Warm-up") | **100084** | |
| #3 | | AB ("Jump" or "Counter Movement Jump" or "CMJ" or "Vertical Jump" or "Squat Jump" or "Drop Jump" or "Abalakov Jump" or "Bosco Repeat Vertical Jump" or "Standing long jump" or "Standing broad jump" or "Power" or "Maximum Output" or "Maximum Output" or "Peak Strength" or "Peak Output" or "Muscle power") | **5695459** | |
| #4 | | #3 AND #2 AND #1 | **453** | |

| Database | Embase | |
| --- | --- | --- |
| Search Date | May 29th, 2025 | |
| Search Period | From the inception of database to May 29th, 2025 | |
| No. | Search strategy | Literatures retrieved |
| #1 | 'squat':ti,ab,kw OR 'barbell squat':ti,ab,kw OR 'back squat':ti,ab,kw OR 'low bar squat':ti,ab,kw OR 'high bar squat':ti,ab,kw OR 'complex training':ti,ab,kw OR 'contrast training':ti,ab,kw OR 'hybrid power training':ti,ab,kw OR 'flywheel eccentric training':ti,ab,kw OR 'flywheel inertial training':ti,ab,kw OR 'flywheel training':ti,ab,kw OR 'flywheel resistance training':ti,ab,kw OR 'inertia flywheel':ti,ab,kw OR 'eccentric overload':ti,ab,kw OR 'isoinertial':ti,ab,kw OR 'deadlift':ti,ab,kw OR 'dead-lift':ti,ab,kw OR 'conventional deadlift':ti,ab,kw OR 'sumo deadlift':ti,ab,kw OR 'romanian deadlift':ti,ab,kw OR 'hex bar deadlift':ti,ab,kw OR 'trap bar deadlift':ti,ab,kw OR 'barbell deadlift':ti,ab,kw OR 'hip hinge exercise':ti,ab,kw OR 'weightlifting deadlift':ti,ab,kw OR 'leg press':ti,ab,kw OR 'lp':ti,ab,kw OR 'leg-press':ti,ab,kw OR 'seated leg press':ti,ab,kw OR '45-degree leg press':ti,ab,kw OR 'horizontal leg press':ti,ab,kw OR 'vertical leg press':ti,ab,kw OR 'pneumatic leg press':ti,ab,kw OR 'machine leg press':ti,ab,kw | **56300** |
| #2 | 'acute responses':ti,ab,kw OR 'acute effects':ti,ab,kw OR 'post-activation potentiation':ti,ab,kw OR 'post-activation performance enhancement':ti,ab,kw OR 'postactivation potentiation':ti,ab,kw OR 'postactivation performance enhancement':ti,ab,kw OR 'pape':ti,ab,kw OR 'pap':ti,ab,kw OR 'warm-up':ti,ab,kw | **65504** |
| #3 | 'jump':ti,ab,kw OR 'counter movement jump':ti,ab,kw OR 'cmj':ti,ab,kw OR 'vertical jump':ti,ab,kw OR 'squat jump':ti,ab,kw OR 'drop jump':ti,ab,kw OR 'abalakov jump':ti,ab,kw OR 'bosco repeat vertical jump':ti,ab,kw OR 'standing long jump':ti,ab,kw OR 'standing broad jump':ti,ab,kw OR 'power':ti,ab,kw OR 'maximum output':ti,ab,kw OR 'peak strength':ti,ab,kw OR 'peak output':ti,ab,kw OR 'muscle power':ti,ab,kw | **517663** |
| #4 | #3 AND #2 AND #1 | **400** |

| Database | Cochrane | |
| --- | --- | --- |
| Search Date | May 29th, 2025 | |
| Search Period | From the inception of database to May 29th, 2025 | |
| No. | Search strategy | Literatures retrieved |
| #1 | "Squat" or "Barbell Squat" or "Back Squat" or "Low Bar Squat" or "High Bar Squat" or "Complex training" or "Contrast training" or " Hybrid Power Training" or "Flywheel eccentric training" or "Flywheel inertial training" or "Flywheel training" or "Flywheel resistance training" or "Inertia Flywheel" or "Eccentric overload" or "Isoinertial" or "deadlift" or "dead-lift" or "Conventional deadlift" or "Sumo deadlift" or "Romanian deadlift" or "Hex bar deadlift" or "Trap bar deadlift" or "Barbell deadlift" or "Hip hinge exercise" or "Weightlifting deadlift" or "leg press" or "LP" or "Leg-press" or "Seated leg press" or "45-degree leg press" or "Horizontal leg press" or "Vertical leg press" or "Pneumatic leg press" or "Machine leg press" in Title Abstract Keyword - (Word variations have been searched) | **9340** |
| #2 | "Acute responses" or "Acute Effects" or "post-activation potentiation" or "post-activation performance enhancement" or "Postactivation Potentiation" or "postactivation performance enhancement" or "PAPE" or "PAP" or "Warm-up" in Title Abstract Keyword - (Word variations have been searched) | **15248** |
| #3 | "Jump" or "Counter Movement Jump" or "CMJ" or "Vertical Jump" or "Squat Jump" or "Drop Jump" or "Abalakov Jump" or "Bosco Repeat Vertical Jump" or "Standing long jump" or "Standing broad jump" or "Power" or "Maximum Output" or "Maximum Output" or "Peak Strength" or "Peak Output" or "Muscle power" in Title Abstract Keyword - (Word variations have been searched) | **67773** |
| #4 | #3 AND #2 AND #1 |  |

| Database | Scopus | |
| --- | --- | --- |
| Search Date | May 29th, 2025 | |
| Search Period | From the inception of database to May 29th, 2025 | |
| No. | Search strategy | Literatures retrieved |
| #1 | TITLE-ABS-KEY ( "Squat" OR "Barbell Squat" OR "Back Squat" OR "Low Bar Squat" OR "High Bar Squat" OR "Complex training" OR "Contrast training" OR " Hybrid Power Training" OR "Flywheel eccentric training" OR "Flywheel inertial training" OR "Flywheel training" OR "Flywheel resistance training" OR "Inertia Flywheel" OR "Eccentric overload" OR "Isoinertial" OR "deadlift" OR "dead-lift" OR "Conventional deadlift" OR "Sumo deadlift" OR "Romanian deadlift" OR "Hex bar deadlift" OR "Trap bar deadlift" OR "Barbell deadlift" OR "Hip hinge exercise" OR "Weightlifting deadlift" OR "leg press" OR "LP" OR "Leg-press" OR "Seated leg press" OR "45-degree leg press" OR "Horizontal leg press" OR "Vertical leg press" OR "Pneumatic leg press" OR "Machine leg press" ) | **116599** |
| #2 | TITLE-ABS-KEY ( "Acute responses" OR "Acute Effects" OR "post-activation potentiation" OR "post-activation performance enhancement" OR "Postactivation Potentiation" OR "postactivation performance enhancement" OR "PAPE" OR "PAP" OR "Warm-up" ) | **75487** |
| #3 | TITLE-ABS-KEY ( "Jump" OR "Counter Movement Jump" OR "CMJ" OR "Vertical Jump" OR "Squat Jump" OR "Drop Jump" OR "Abalakov Jump" OR "Bosco Repeat Vertical Jump" OR "Standing long jump" OR "Standing broad jump" OR "Power" OR "Maximum Output" OR "Maximum Output" OR "Peak Strength" OR "Peak Output" OR "Muscle power" ) | **4785072** |
| #4 | #3 AND #2 AND #1 | **677** |
