# Supplementary material for: Acute enhancement strategies for countermovement jump performance: a network meta-analysis of different resistance training protocols
Source: Front Physiol. 2026 Jan 30;17:1729372. doi: 10.3389/fphys.2026.1729372 (PMC12902682; doi:10.3389/fphys.2026.1729372)

Supplementary Appendix

Acute Enhancement Strategies for Vertical Jump Performance A Network Meta-Analysis of Different Resistance Training Protocols

Table of contents

Appendix 1: Risk of bias of randomized clinical trials.............................2

Appendix 2: Evaluation of inconsistency and heterogeneity.................... 8

Appendix 3: Network maps and forest plots of secondary outcomes.......9

Appendix 4: SUCRA and cumulative probability plots.......................... 10

Appendix5: Ranking List of Comprehensive Evaluation Results of Different Resistance Training Methods on Vertical Jump Ability Based on Network Meta-analysis.. 12

Appendix 6： Subgroup analysis of heterogeneity............................... 13

Appendix 7： Sensitivity Analysis...16

Appendix 1: Risk of bias of randomized clinical trials

Figure 1-1 The risk of bias for each trial

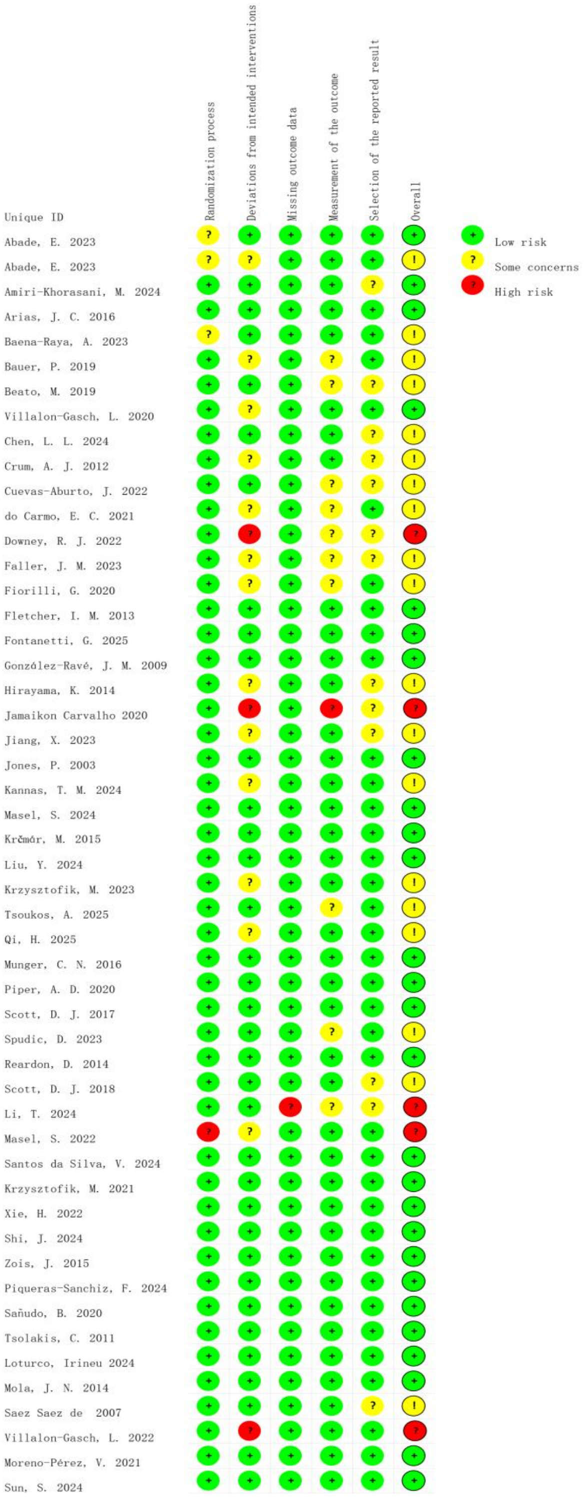

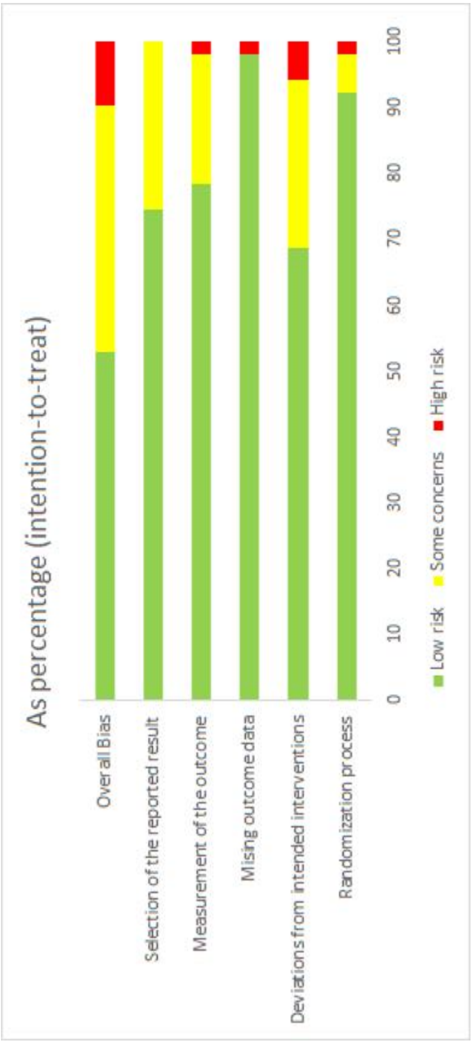

Figure 1-2 As percentage (intention-to-treat) of ROB2

	Randomization process	Deviations from intended interventions	Mising outcome data	Measurement of the outcome	Selection of the reported result	Overall Bias
Assignment to intervention (the 'intention-to-treat' effect)						
Total number of study = 51						
Low risk	92.2	68.6	98	78.4	74.5	52.9
Some concerns	5.9	25.5	0	19.6	25.5	37.3
High risk	2	5.9	2	2	0	9.8

Table 1 Study level risk of bias assessment using Cochrane risk of bias tool 2.0 for assessing risk of bias of randomized clinical trials.

Unique ID	Randomization process	Deviations from intended interventions	Mising outcome data	Measurement of the outcome	Selection of the reported result	Overall Bias
Abade, E. 2023	Some concerns	Low	Low	Low	Low	Low
Amiri-Khorasani, M. 2024	Some concerns	Some concerns	Low	Low	Low	Some concerns
Arias, J. C. 2016	Low	Low	Low	Low	Some concerns	Low
Baena-Raya, A. 2023	Low	Low	Low	Low	Low	Low
Bauer, P. 2019	Some concerns	Low	Low	Low	Low	Some concerns
Beato, M. 2019	Low	Some concerns	Low	Some concerns	Low	Some concerns
Villalon-Gasch, L. 2020	Low	Low	Low	Some concerns	Some concerns	Some concerns
Chen, L. L. 2024	Low	Some concerns	Low	Low	Low	Low
Crum, A. J. 2012	Low	Low	Low	Low	Some concerns	Some concerns
Cuevas-Aburto, J. 2022	Low	Some concerns	Low	Low	Some concerns	Some concerns
do Carmo, E. C. 2021	Low	Low	Low	Some concerns	Some concerns	Some concerns
Downey, R. J. 2022	Low	Some concerns	Low	Some concerns	Low	Some concerns
Faller, J. M. 2023	Low	High	Low	Some concerns	Some concerns	High
Fiorilli, G. 2020	Low	Some concerns	Low	Some concerns	Some concerns	Some concerns
Fletcher, I. M. 2013	Low	Some concerns	Low	Some concerns	Low	Some concerns
Fontanetti, G. 2025	Low	Low	Low	Low	Low	Low
González-Ravé, J. M. 2009	Low	Low	Low	Low	Low	Low
Hirayama, K. 2014	Low	Low	Low	Low	Low	Low
Jamaikon Carvalho 2020	Low	Some concerns	Low	Low	Some concerns	Some concerns
Jiang, X. 2023	Low	High	Low	High	Some concerns	High
Jones, P. 2003	Low	Some concerns	Low	Low	Some concerns	Some concerns

Kannas, T. M. 2024	Low	Low	Low	Low	Low	Low
Masel, S. 2024	Low	Some concerns	Low	Low	Low	Some concerns
Krčmár, M. 2015	Low	Low	Low	Low	Low	Low
Liu, Y. 2024	Low	Low	Low	Low	Low	Low
Krzysztofik, M. 2023	Low	Low	Low	Low	Low	Low
Tsoukos, A. 2025	Low	Some concerns	Low	Low	Low	Some concerns
Qi, H. 2025	Low	Low	Low	Some concerns	Low	Some concerns
Munger, C. N. 2016	Low	Some concerns	Low	Low	Low	Some concerns
Piper, A. D. 2020	Low	Low	Low	Low	Low	Low
Scott, D. J. 2017	Low	Low	Low	Low	Low	Low
Spudic, D. 2023	Low	Low	Low	Low	Low	Low
Reardon, D. 2014	Low	Low	Low	Some concerns	Low	Some concerns
Scott, D. J. 2018	Low	Low	Low	Low	Low	Low
Li, T. 2024	Low	Low	Low	Low	Some concerns	Some concerns
Masel, S. 2022	Low	Low	High	Some concerns	Some concerns	High
Santos da Silva, V. 2024	High	Some concerns	Low	Low	Low	High
Krzysztofik, M. 2021	Low	Low	Low	Low	Low	Low
Xie, H. 2022	Low	Low	Low	Low	Low	Low
Shi, J. 2024	Low	Low	Low	Low	Low	Low
Zois, J. 2015	Low	Low	Low	Low	Low	Low
Piqueras-Sanchiz, F. 2024	Low	Low	Low	Low	Low	Low
Sañudo, B. 2020	Low	Low	Low	Low	Low	Low
Tsolakis, C. 2011	Low	Low	Low	Low	Low	Low
Loturco, Irineu 2024	Low	Low	Low	Low	Low	Low
Mola, J. N. 2014	Low	Low	Low	Low	Low	Low

Saez Saez de 2007	Low	Low	Low	Low	Low	Low
Villalon-Gasch, L. 2022	Low	Low	Low	Low	Some concerns	Some concerns
Moreno-Pérez, V. 2021	Low	High	Low	Low	Low	High
Sun, S. 2024	Low	Low	Low	Low	Low	Low
Timon, R. 2019	Low	Low	Low	Low	Low	Low

Figure 1-3 Funnel Chart

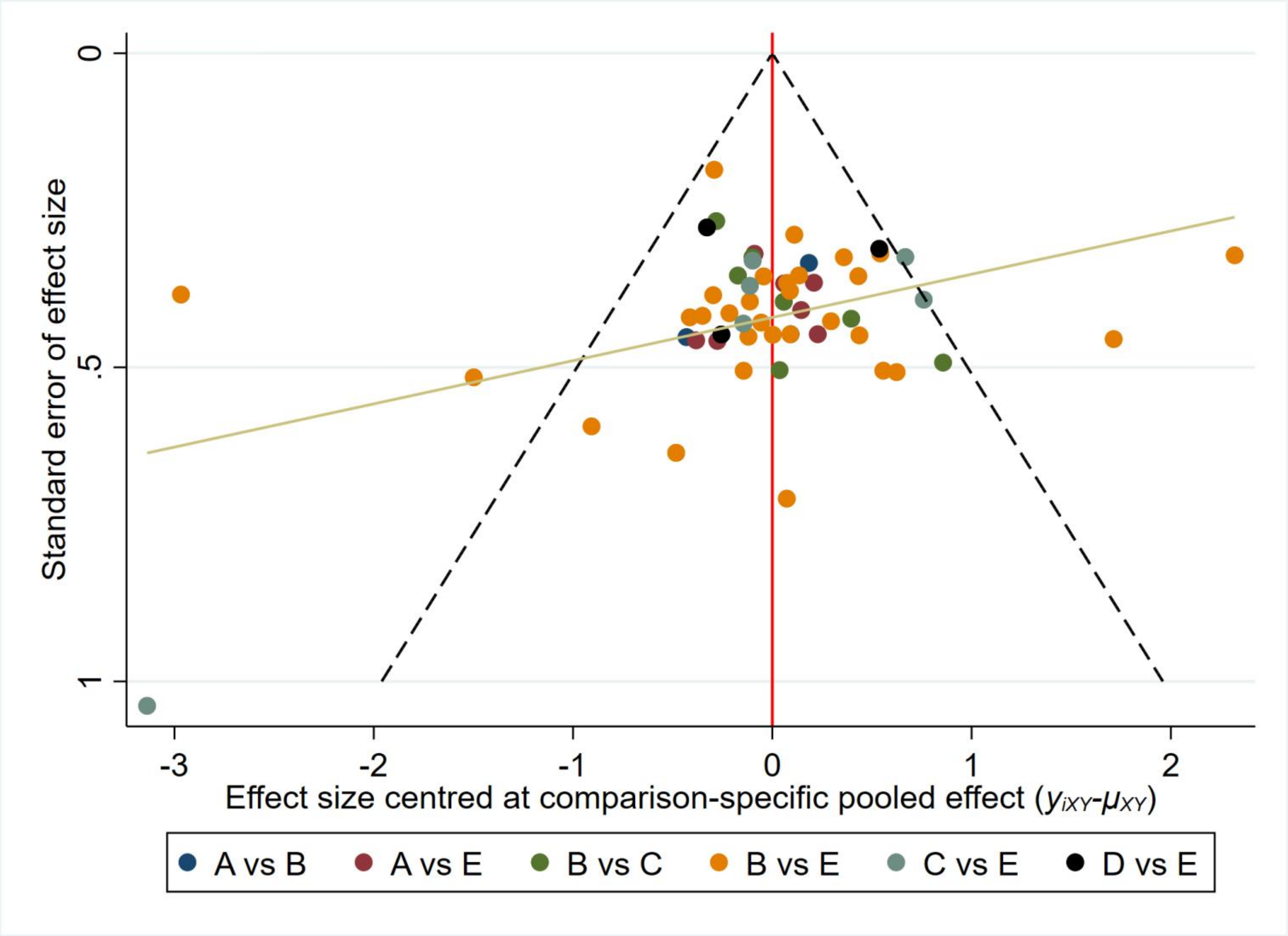

Appendix 2: Evaluation of inconsistency and heterogeneity

Figure 2-1 Global inconsistency test

```
( 3)  [_y_E]des_BCE = 0
( 4)  [_y_C]des_BCE = 0
( 5)  [_y_E]des_BE = 0
( 6)  [_y_C]des_CE = 0

          chi2(  6) =    1.22
        Prob > chi2 =    0.9758
vmeta command stored as F9; test comman
```

Figure 2-2 Figure Consistency (heterogeneity) test

Estimated between-studies SDs and correlation matrix

	SD	_y_B	_y_C	_y_D	_y_E
_y_B	.63744566	1	.	.	.
_y_C	.63744566	.5	1	.	.
_y_D	.63744566	.5	.5	1	.
_y_E	.63744566	.5	.5	.5	1

mvmeta command stored as F9

Figure 2-3 Node-splitting method

Side	Direct		Indirect		Difference			tau
	Coef.	Std. Err.	Coef.	Std. Err.	Coef.	Std. Err.	P>|z|	
A B	-.0726193	.4749309	-.1470126	.426448	.0743933	.6380965	0.907	[illegible]
A E	-.3355202	.3353612	-.1542741	.6694958	-.1812461	.7486717	0.809	[illegible]
B C	.4160634	.3344222	.3280658	.7139914	.0879976	.7884629	0.911	[illegible]
B E	-.1790764	.1735803	-.2554014	.5782498	.076325	.6037866	0.899	[illegible]
C E	-.4127747	.5540098	-.6759885	.4009962	.2632138	.6838964	0.700	[illegible]
D E *	.0403804	.7522152	-.6382061	632.3667	.6785865	632.3672	0.999	[illegible]

Appendix 3: Network maps and forest plots of secondary outcomes

Figure 3-1 The networked data graph of vertical jump ability

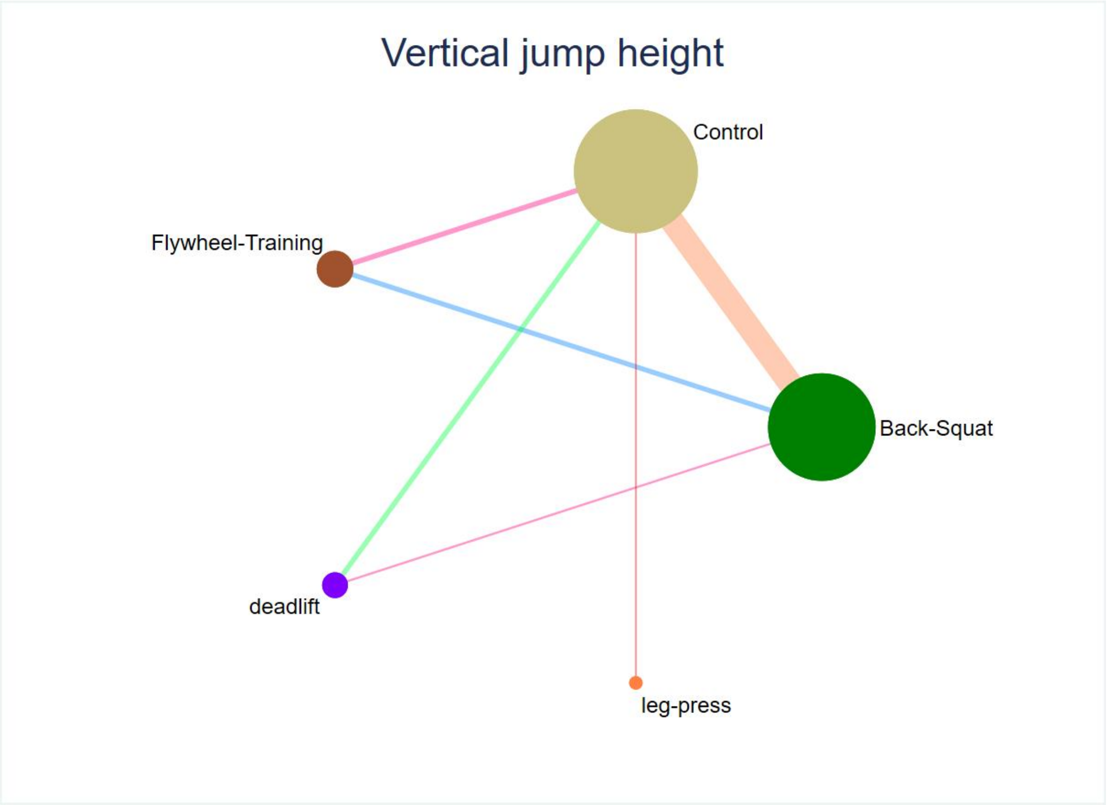

Figure 3-2 Network forest

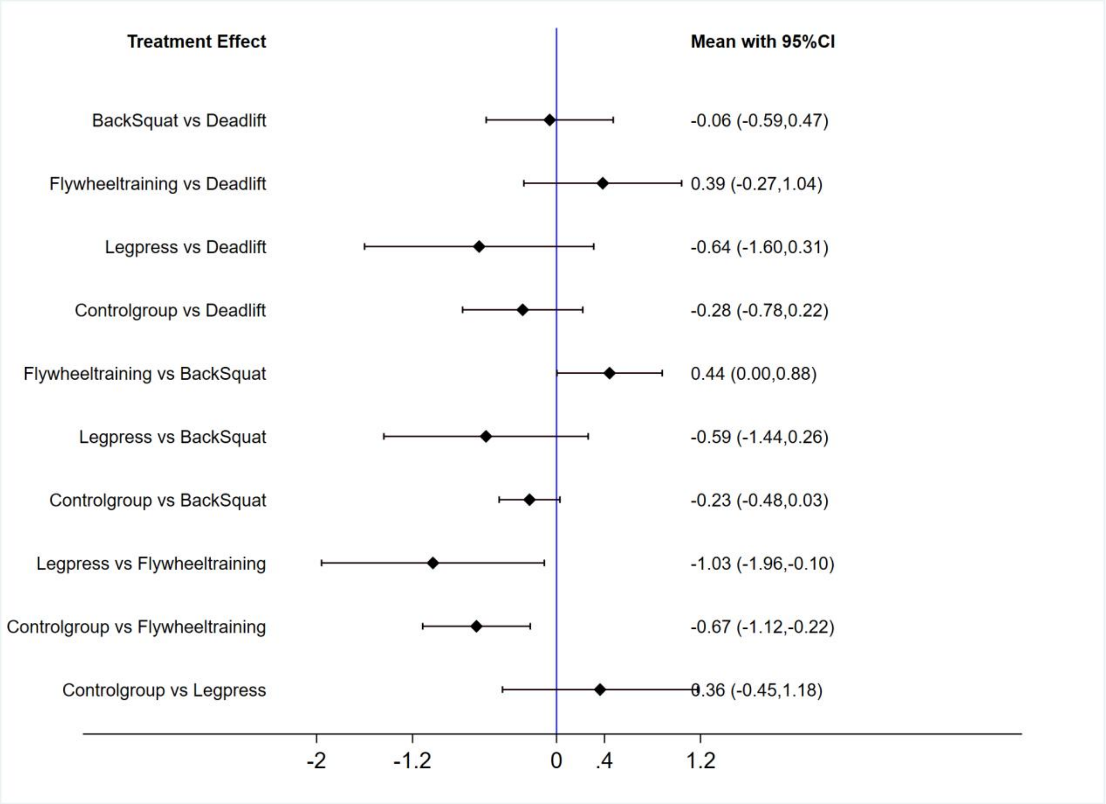

Appendix 4:SUCRA and cumulative probability plots

Figure 4-1 Cumulative probability graph（1）

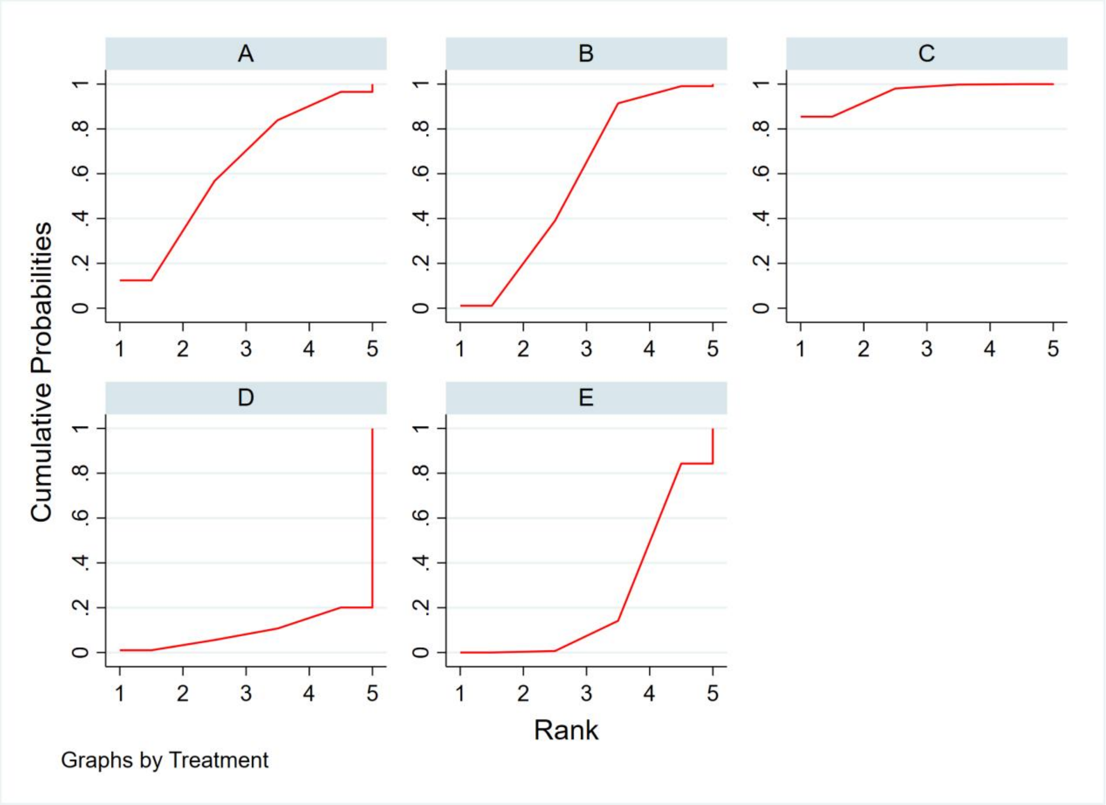

Figure 4-2 Cumulative probability graph(2)

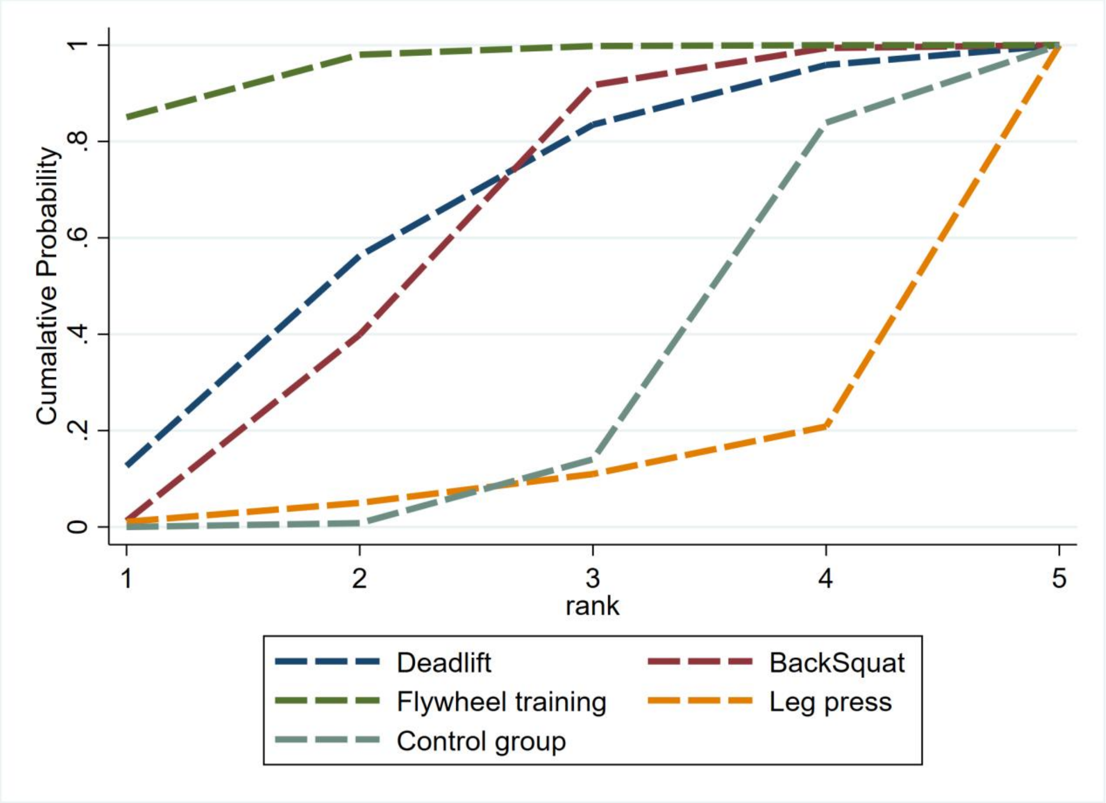

Appendix 5 : Ranking List of Comprehensive Evaluation Results of Different Resistance Training Methods on Vertical Jump Ability Based on Network Meta-analysis

Figure 5-1 League Table

_A_	_B_	_C_	_D_	_E_
**A**	-0.06 (-0.59, 0.47)	0.39 (-0.27, 1.04)	-0.64 (-1.60, 0.31)	-0.28 (-0.78, 0.22)
0.06 (-0.47, 0.59)	**B**	0.44 (0.00, 0.88)	-0.59 (-1.44, 0.26)	-0.23 (-0.48, 0.03)
-0.39 (-1.04, 0.27)	-0.44 (-0.88, -0.00)	**C**	-1.03 (-1.96, -0.10)	-0.67 (-1.12, -0.22)
0.64 (-0.31, 1.60)	0.59 (-0.26, 1.44)	1.03 (0.10, 1.96)	D	0.36 (-0.45, 1.18)
0.28 (-0.22, 0.78)	0.23 (-0.03, 0.48)	0.67 (0.22, 1.12)	-0.36 (-1.18, 0.45)	**E**

A	deadlift
B	BackSquat
C	Flywheeltraining
D	legpress
C	Control group

Figure 5-2 SUCRA Sorting Chart

Treatm~t	SUCRA	PrBest	MeanRank
A	62.4	12.4	2.5
B	57.6	1.1	2.7
C	95.8	85.5	1.2
D	9.4	1.0	4.6
E	24.8	0.0	4.0

Appendix 6 Subgroup analysis of heterogeneity

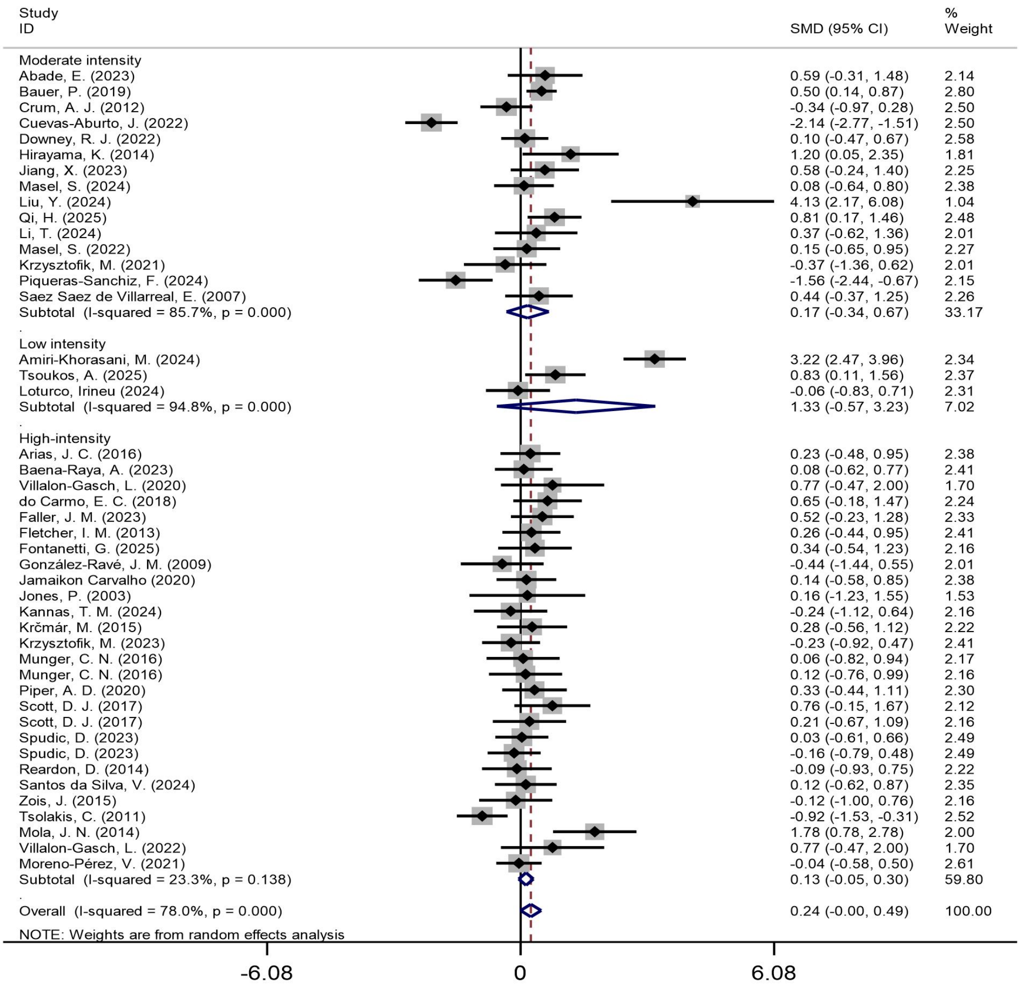

Figure 6-1 Subgroup analysis of training intensity for heterogeneity（Forest plot）

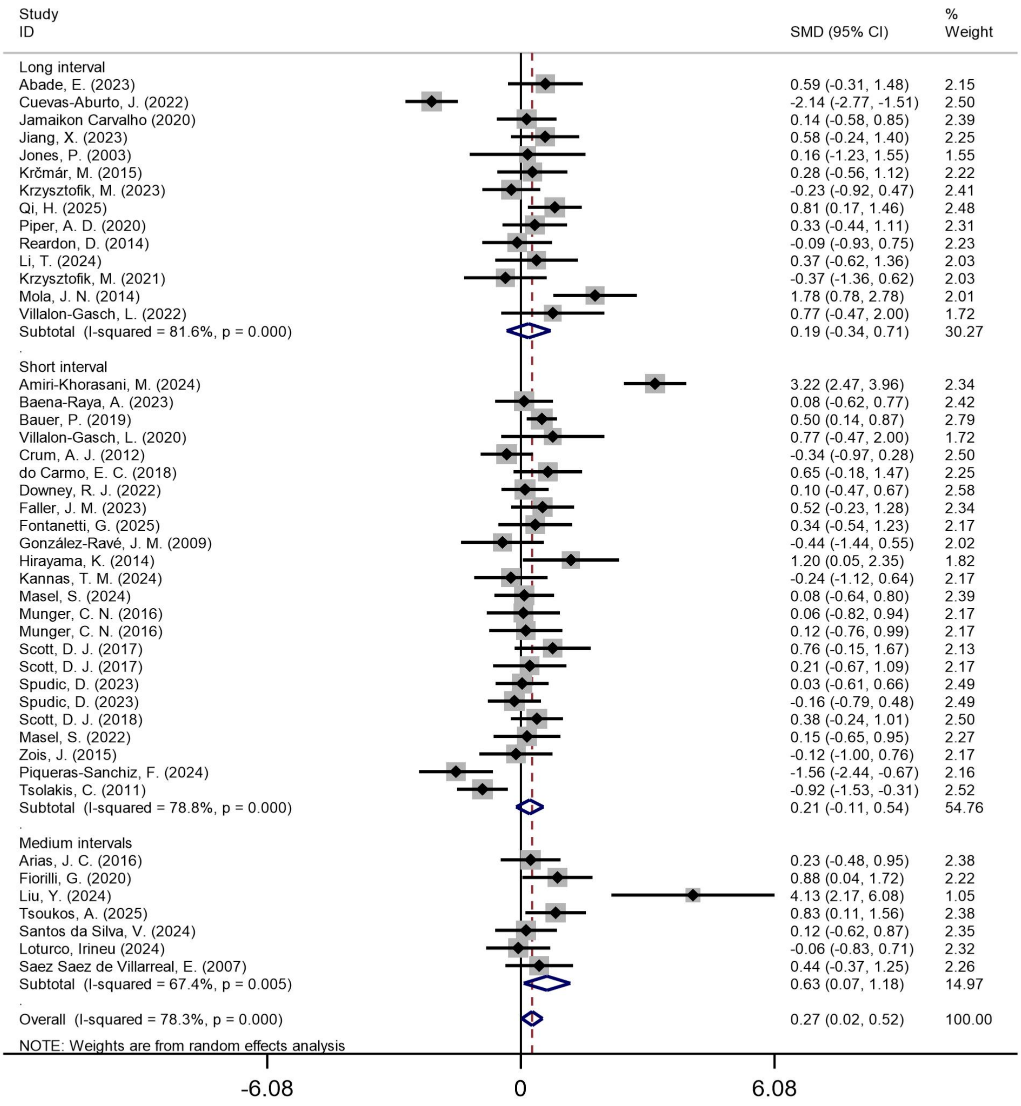

Figure 6-2 Subgroup analysis of the intermittent time for heterogeneity（Forest plot）

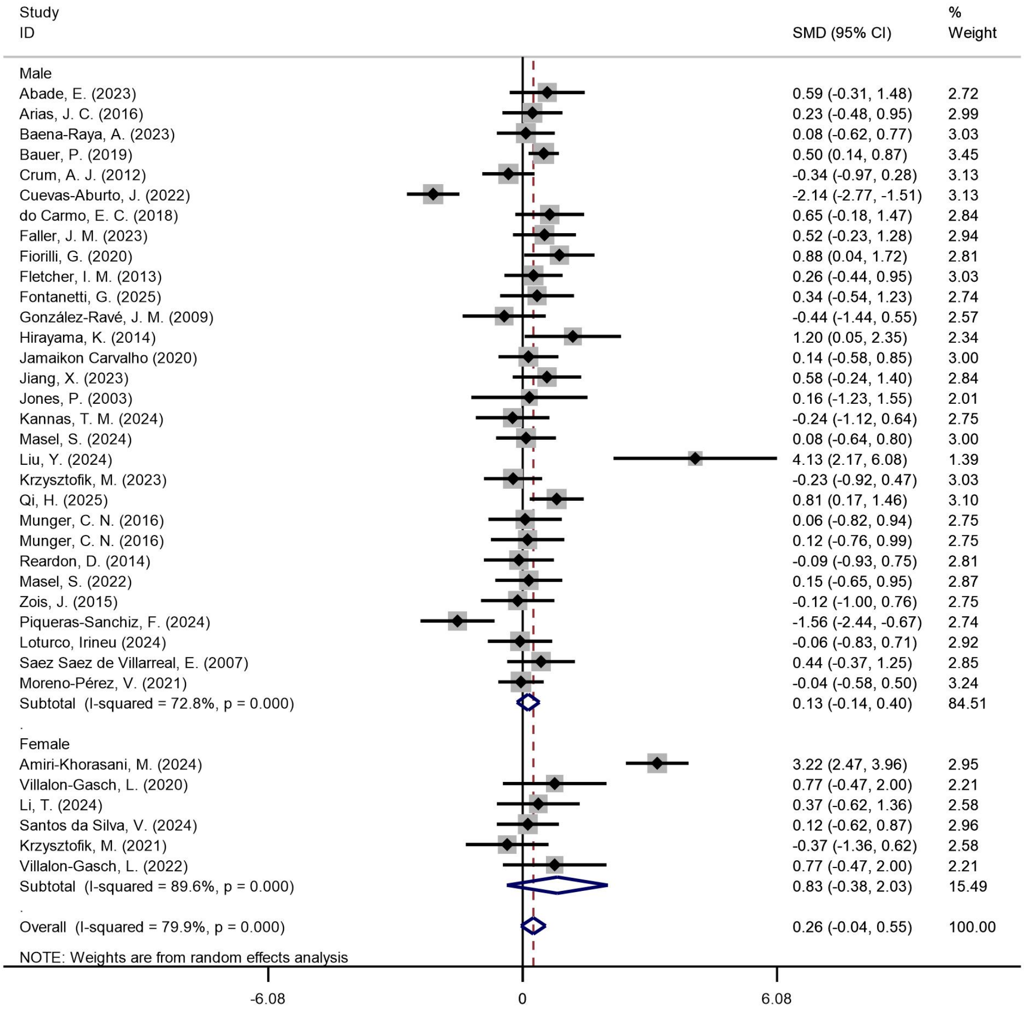

Figure 6-3 Subgroup analysis of the gender for heterogeneity（Forest plot）

Appendix 7 Sensitivity Analysis(A sensitivity analysis was performed using only the data from the final post-intervention time point.)

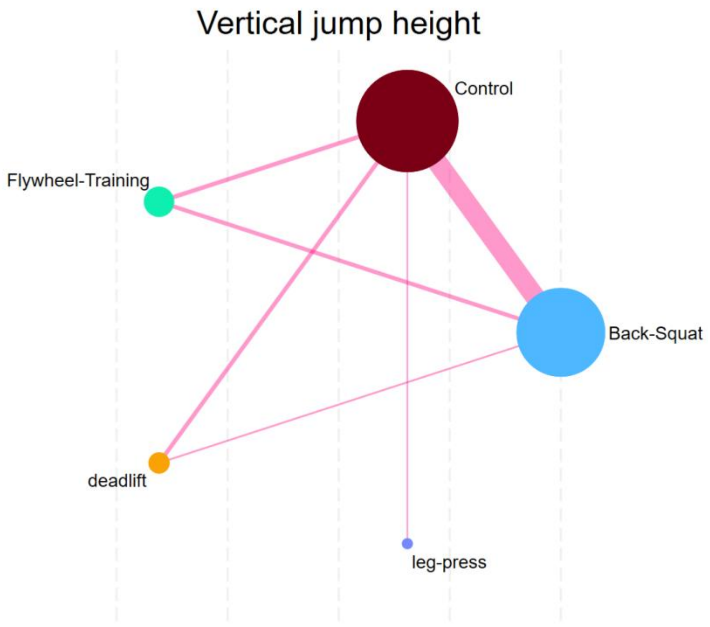

Figure 7-1 Network diagram of the effects of different resistance training on vertical jump ability

```
Testing for inconsistency:
 ( 1)  [_y_B]des_ABC = 0
 ( 2)  [_y_B]des_ABD = 0
 ( 3)  [_y_C]des_AC = 0
 ( 4)  [_y_D]des_AD = 0
 ( 5)  [_y_C]des_BC = 0
 ( 6)  [_y_D]des_BD = 0

           chi2(  6) =    1.42
         Prob > chi2 =    0.9649
mvmeta command stored as F9; test command stored as F8
```

Figure 7-2 Testing for inconsistency

```
Multivariate meta-analysis
Variance-covariance matrix = proportional .5*I(4)+.5*J(4,4,1)
Method = reml                                 Number of dimensions    =     4
Restricted log likelihood = -132.92301        Number of observations  =    51

             Coefficient  Std. err.      z    P>|z|     [95% conf. interval]

_y_B
       _cons   -.1596722    .124735    -1.28   0.201    -.4041483    .0848039

_y_C
       _cons    .4640751     .21662     2.14   0.032     .0395078    .8886424

_y_D
       _cons    .1136936   .2615323     0.43   0.664    -.3989002    .6262874

_y_E
       _cons   -.5230369   .4192335    -1.25   0.212     -1.34472    .2986457

Estimated between-studies SDs and correlation matrix

             SD        _y_B        _y_C        _y_D        _y_E
_y_B  .60126333          1           .           .           .
_y_C  .60126333         .5           1           .           .
_y_D  .60126333         .5          .5           1           .
_y_E  .60126333         .5          .5          .5           1

mvmeta command stored as F9
```

Notes: τ 2=0.6^2=0.36

Figure 7-3 Consistency test

Table 7-1 League table of network comparisons of the effects of Different Resistance Training Methods on Vertical Jump Performance

**leg press**	-0.54 (-1.40; 0.31)	-0.66 (-1.62; 0.31)	-1.05 (-1.98; -0.11)	-0.37 (-1.19; 0.45)
-0.54 (-1.40; 0.31)	**Back Squat**	-0.11 (-0.65; 0.42)	-0.50 (-0.94; -0.06)	0.17 (-0.08; 0.43)
-0.66 (-1.62; 0.31)	-0.11 (-0.65; 0.42)	**deadlift**	-1.05 (-1.98; -0.11)	0.29 (-0.22; 0.79)
-1.05 (-1.98; -0.11)	-0.50 (-0.94; -0.06)	-0.39 (-1.05; 0.27)	**Flywheel training**	0.68 (0.23;1.12)
-0.37 (-1.19; 0.45)	0.17 (-0.08; 0.43)	0.29 (-0.22; 0.79)	0.68 (0.23; 1.12)	**Control group**

Table 7-2 Ranking of intervention effects

Treatmt	SUCRA	PrBest	MeanRank
A	53. 4	0. 6	2. 9
B	26. 5	0	3. 9
C	95. 7	84. 7	1. 2
D	64. 4	13. 6	2. 4
E	9. 9	1. 1	4. 6

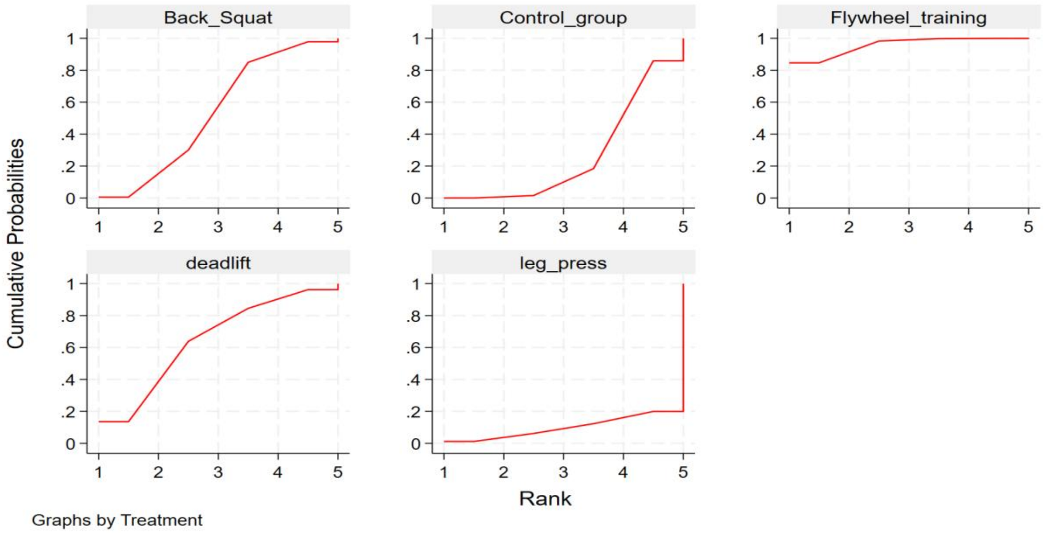

Figure 7-5 SUCRA Sorting Chart

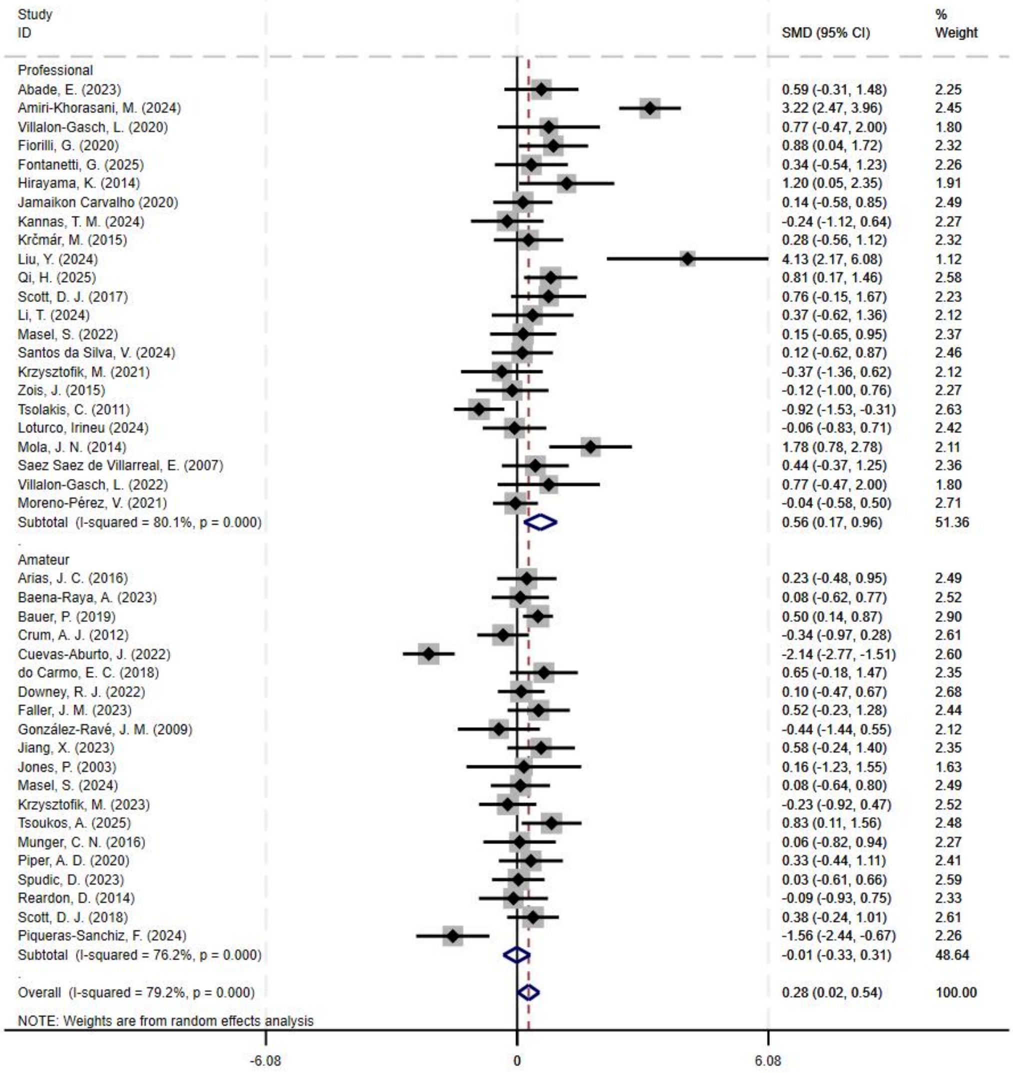

Figure 7-6 Subgroup analysis (professional status)

```
. db metareg

. metareg _ES 亚组, wsse(_seES) bsest(reml)

Meta-regression                                    Number of obs   =      43
REML estimate of between-study variance            tau2            =   .5436
% residual variation due to heterogeneity          I-squared_res   =  78.51%
Proportion of between-study variance explained     Adj R-squared   =   8.29%
With Knapp-Hartung modification
```

_ES	Coefficient	Std. err.	t	P>|t|	[95% conf. interval]	
亚组	-.5610224	.2680135	-2.09	0.043	-1.102287	-.0197581
_cons	1.111603	.4204386	2.64	0.012	.2625101	1.960696

Figure 7-7 Meta regression (professional status)

Figure 7-8 Subgroup analysis (Baseline Strength Level)

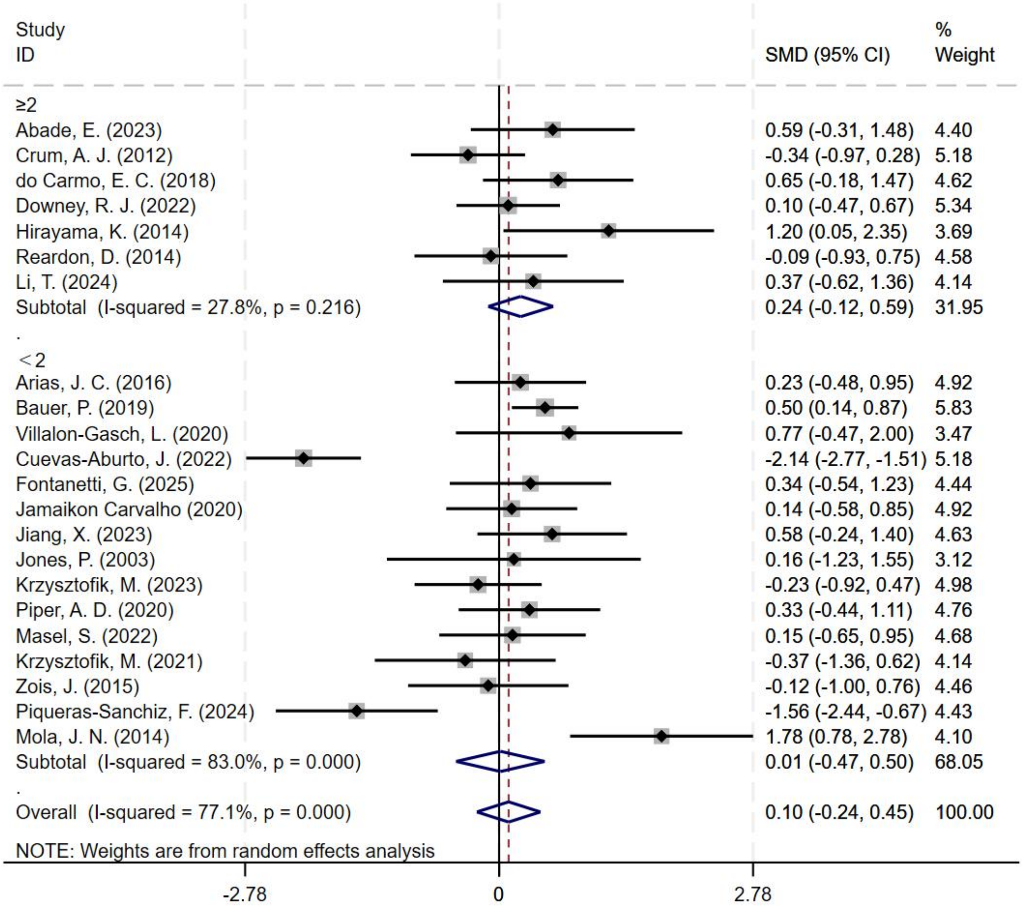

Figure 7-9 Meta regression (Baseline Strength Level)

```
. db metareg

. metareg _ES 亚组, wsse(_seES) bsest(reml)

Meta-regression                                     Number of obs  =        22
REML estimate of between-study variance             tau2           =     .5121
% residual variation due to heterogeneity           I-squared_res  =    77.93%
Proportion of between-study variance explained      Adj R-squared  =    -2.84%
With Knapp-Hartung modification
------------------------------------------------------------------------------
         _ES | Coefficient  Std. err.      t    P>|t|     [95% conf. interval]
-------------+----------------------------------------------------------------
        亚组 |   .3024113   .3822394     0.79   0.438    -.4949262    1.099749
       _cons |  -.2944375   .5349241    -0.55   0.588     -1.41027    .8213946
------------------------------------------------------------------------------
```

Figure 7-10 Subgroup analysis (Training Experience)

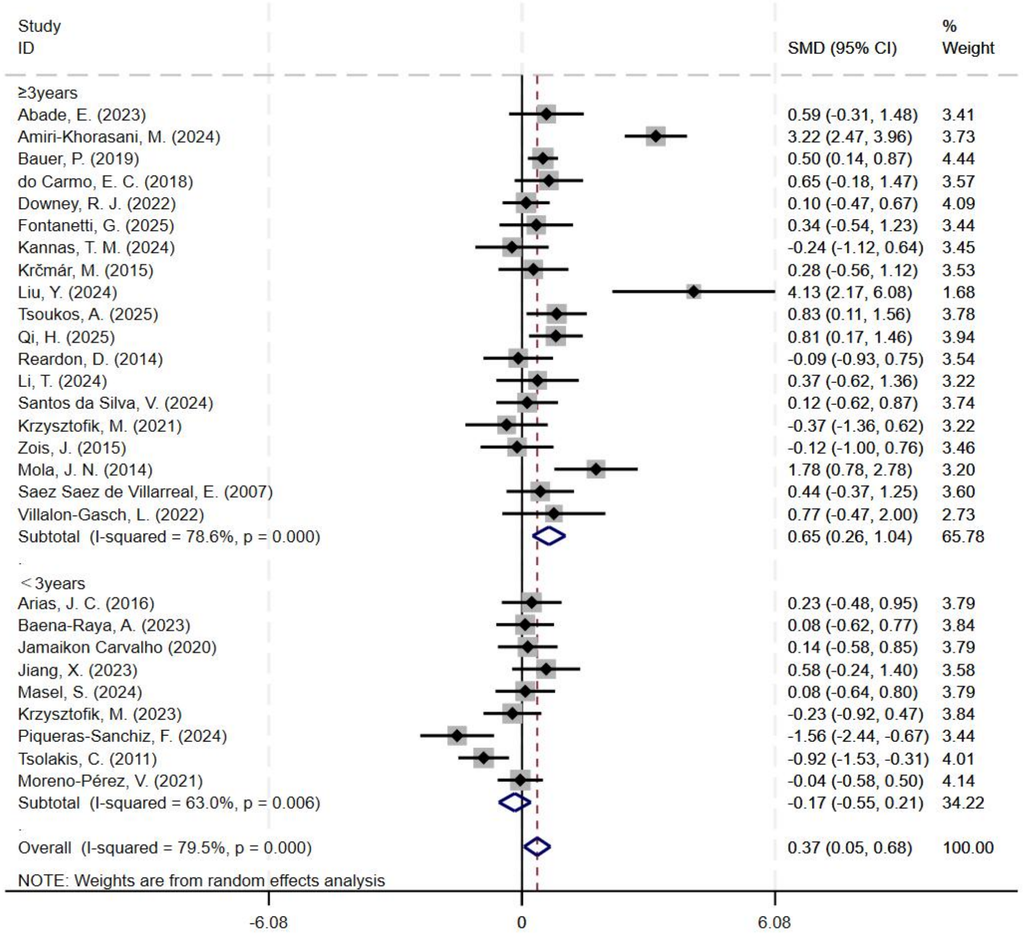

Figure 7-11 Meta regression (Training Experience)

```
. metareg _ES 亚组, wsse(_seES) bsest(reml)

Meta-regression                                    Number of obs    =       28
REML estimate of between-study variance            tau2             =    .5212
% residual variation due to heterogeneity          I-squared_res    =   75.40%
Proportion of between-study variance explained     Adj R-squared    =   21.16%
With Knapp-Hartung modification
```

_ES	Coefficient	Std. err.	t	P>|t|	[95% conf.	interval]
亚组	.8220778	.3572652	2.30	0.030	.0877087	1.556447
_cons	-.9976234	.615833	-1.62	0.117	-2.263486	.2682395

Figure 7-12 Exclude the sucra values that include the results of the SJ study

```
. sucra prob*, labels(Back_Squat Control_group Flywheel_training deadlift leg_press) lcol(red black)

Treatment Relative Ranking of Model 1
```

Treatment	SUCRA	PrBest	MeanRank
Back_Squat	57.0	1.2	2.7
Control_group	24.6	0.0	4.0
Flywheel_training	95.0	83.0	1.2
deadlift	63.4	14.6	2.5
leg_press	10.0	1.2	4.6

Figure 7-13 Exclude the SUCRA curve that includes the results of the SJ study

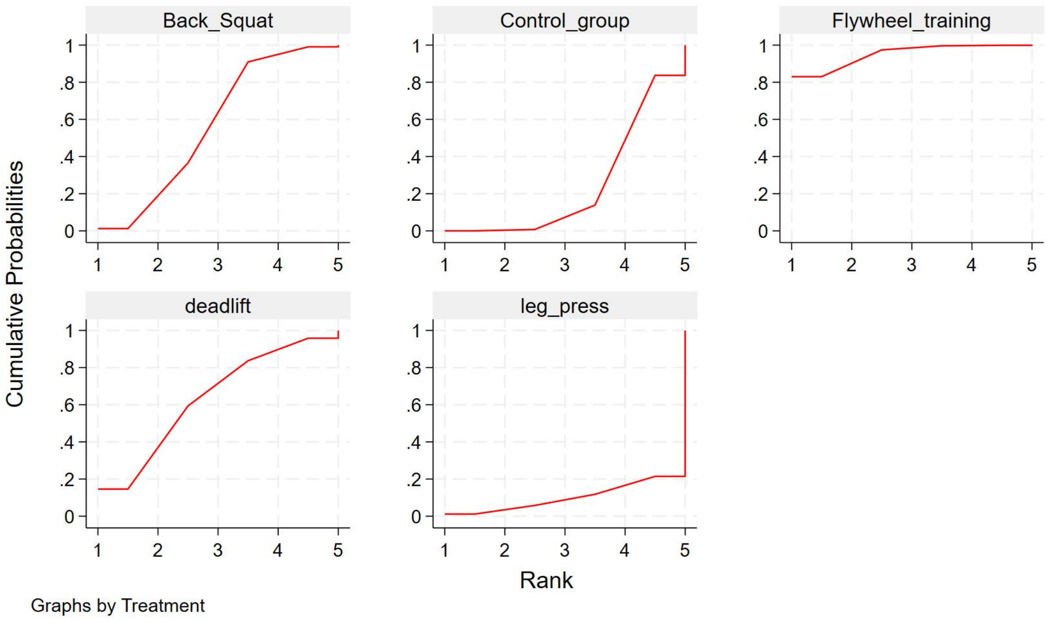

Supplement: Supplementary file 3 [file Supplementaryfile2.pdf]
